# Heterologous Prime-Boost Vaccination with a Peptide-Based Vaccine and Viral Vector Reshapes Dendritic Cell, CD4+ and CD8+ T Cell Phenotypes to Improve the Antitumor Therapeutic Effect

**DOI:** 10.3390/cancers13236107

**Published:** 2021-12-03

**Authors:** Tamara Hofer, Matteo Rossi, Susanna Carboni, Wilma Di Berardino Besson, Dorothee von Laer, Guido Wollmann, Madiha Derouazi, Marie-Laure Santiago-Raber

**Affiliations:** 1 Christian Doppler Laboratory for Viral Immunotherapy of Cancer, Medical University of Innsbruck, Peter-Mayr-Straße 4b, 6020 Innsbruck, Austria; tamara.hofer@i-med.ac.at (T.H.); guido.wollmann@i-med.ac.at (G.W.); 2 Division of Virology, Medical University of Innsbruck, Peter-Mayr-Straße 4b, 6020 Innsbruck, Austria; dorothee.von-laer@i-med.ac.at; 3 AMAL Therapeutics, Fondation Pour Recherches Médicales, Avenue de la Roseraie 64, 1205 Geneva, Switzerland; matteo.rossi@boehringer-ingelheim.com (M.R.); susanna.carboni@boehringer-ingelheim.com (S.C.); wilma.besson-di_berardino@boehringer-ingelheim.com (W.D.B.B.); 4 Boehringer Ingelheim International GmbH, 55216 Ingelheim, Germany

**Keywords:** therapeutic prime-boost vaccine, t-helper 1 cells, cross-presenting dendritic cells, cold and hot tumor models, antigen competition, multi-epitope vaccine

## Abstract

**Simple Summary:**

Developing new therapeutic cancer vaccines is of paramount importance to counteract tumor escape observed after conventional therapies in certain types of cancer. We have previously shown that the combination of two different vaccine platforms, targeting tumor-specific antigens, resulted in potent immune responses in preclinical models. Here, we show that the heterologous prime-boost combination with a protein vaccine and a viral vector vesicular stomatitis virus immunologically reshapes the immune-excluded TC-1 tumor model as well as the inflamed MC-38 tumor model, leading to beneficial therapeutic efficacy. Furthermore, the treatment with a multi-epitope vaccine allowed us to appreciate the various repartition among three antigen-specific cytotoxic T-cell responses combined with the viral boost. The combination leads to improved efficacy in all animals and highlights the importance of combining tumor epitopes. Our vaccine strategy could be further extended to prophylactic cancer vaccines and beyond, for infectious diseases.

**Abstract:**

Heterologous prime-boost settings with a protein vaccine and the viral vector vesicular stomatitis virus, both expressing tumor-associated antigens (KISIMA-TAA and VSV-GP-TAA), have been previously shown to generate potent antitumor immunity. In the cold TC-1 model (HPV antigen) and the immune-infiltrate MC-38 model (Adpgk, Reps1 and Rpl18 neo-antigens), we further investigated pivotal immune cells that educate CD8+ T cells. Heterologous prime-boost vaccination induced a superior antitumor response characterized by the increase in number and functionality of antigen-specific CD8+ T cells, recruitment of cross-presenting dendritic cells, and polarization of CD4+ T cells towards an antitumor Th1 phenotype within the tumor and tumor-draining lymph nodes, turning the cold TC-1 tumor into a hot, inflamed tumor. In the inflamed MC-38 tumor model, treatment combination markedly prolonged the overall survival of mice. Treatment with multi-epitope vaccines also induced high frequencies of multiple antigen specificities in the periphery and in the tumor. Prime-boost treatment reduced tumor-infiltrating regulatory CD4+ T cells whilst increasing cross-presenting dendritic cells in tumor-draining lymph nodes. In conclusion, heterologous prime-boost vaccination possesses the ability to induce a potent anti-tumor response in both immune-excluded and immune-infiltrated mouse tumor models. Additionally, this study highlights the design of a multi-epitope vaccine for cancer immunotherapy.

## 1. Introduction

The established therapeutic cancer vaccine platform, KISIMA^®^, was previously shown to generate protein vaccines able to induce a robust and prolonged immune response. The recombinant fusion protein vaccine construct is composed of (i) a cell-penetrating peptide (CPP) conferring enhanced vaccine delivery, promoting efficient antigen loading allowing presentation on both major histocompatibility complex (MHC) class I and II molecules [1], (ii) a multi-antigenic domain (Mad), rationally designed containing CD8 and CD4 epitopes from different tumor-specific antigens across a range of HLA restrictions and (iii) a TLR2/4 agonist peptide, which confers self-adjuvant activity to the vaccine [2].

KISIMA^®^-derived vaccines have been shown to address different therapeutic vaccination challenges, the combined effect of the CPP and the peptidic TLR2/4 agonist results in simultaneous antigen presentation and activation of antigen-presenting cells (APCs), leading to an efficient multi-antigenic cellular immune response for different HLA restrictions. Helper and cytotoxic T-cell responses were observed against model-, neo- and self-antigens and were highly potent in several murine tumor models [2].

The heterologous prime-boost vaccination strategy has been investigated and was shown to achieve the best results in terms of the induction of long-lived protective CD8+ T cells [3] and is thought to be suitable at improving treatment efficacy while counteracting tumor immune escape in cancer therapy [4].

As a promising viral platform for both oncolytic and vaccine application, vesicular stomatitis virus (VSV)-GP is a modified chimeric variant of VSV in which the surface glycoprotein G was substituted by the LCMV surface glycoprotein (GP). In contrast to wild-type VSV, VSV-GP lacks neurotoxicity and does not readily induce neutralizing antibodies in mice [5]. Preclinically, VSV-GP has been used effectively as a vaccine vector for the ovalbumin model antigen [6] and as a potent vaccine vector (VSV-GP-Fsyn) in a respiratory syncytial virus (RSV) vaccine study [7] or for HIV vaccine [8]. Clinically, VSV-based vaccines have shown their safety and potent immunogenicity against the Ebola virus, recently leading to their approval [9]. As an oncolytic virus, VSV-GP preferentially replicates in type-I Interferon (IFN)-deficient cells [10], found in various tumors [11], leading to cell lysis while virus propagation in normal tissues is suppressed by an intact antiviral IFN response.

We have previously shown that the heterologous combination of KISIMA-TAA with VSV-GP-TAA induces a potent tumor-specific immune response against various epitopes. Compared to the respective homologous vaccination regimen, the prime-boost combination strongly enhanced the quantities and qualities of antigen-specific cytotoxic T cells (CTLs) while also remodeling the tumor microenvironment (TME) towards a pro-inflammatory composition. Furthermore, targeting PD-1/PDL-1 interaction further increased the anti-tumoral efficacy of the heterologous prime-boost vaccination [12].

Here, we show that in addition to the pro-therapeutic TME repolarization, enhanced quantity and improved quality of circulating and intratumoral antigen-specific CTLs, the heterologous combination also impacted T-helper cell response and T-cell priming within the tumor-draining lymph nodes (-dLNs) involving specific APCs, such as cross-presenting dendritic cells (DCs). We address the CD4 T-cell help, the antigen-specific CD8 T-cell response and involved DCs populations in different tissues using two different tumor-mouse models characterized by the oncoviral antigen E7 in the TC-1 model or three neo-antigens in the MC-38 model (Adpgk, Reps1 and Rpl18), where antigen competition can be further addressed in the latter.

## 2. Materials and Methods

### 2.1. Ethics Approval

Animal experiments were conducted respecting the Swiss federal law on animal protection and were reviewed and approved by the cantonal and institutional veterinary authorities on 18 August 2020 (Authorisation # GE/132/20).

### 2.2. Mice

Six to eight-week-old C57BL/6j female mice were purchased from Charles River (L’Arbresles, France) and were housed in individually ventilated cages in a pathogen-free mouse facility. Tumors were measured using the formula (width × length² × π)/6 and were euthanized when reaching a tumor size ≥1000 mm³ or at indicated time points for tissue harvest.

### 2.3. Tumor Implantation

TC-1 tumor cells provided by T.C. Wu (Johns Hopkins University, Maryland, USA) and MC-38 tumor cells (kindly gifted from Gottfried Baier; Medical University of Innsbruck, Innsbruck, Austria) were cultured and implanted as previously described [12].

### 2.4. Generation of Vaccines

Recombinant protein vaccine KISIMA constructs were produced as described [2]. VSV-GP-HPV and KISIMA-Mad25 have been described previously and contain either full-length proteins or epitopes of the HPV-E2, -E6 and E7 proteins [12]. The KISIMA-Mad46 peptide vaccine was generated de novo as described previously [2] and consisted of a CPP, a Mad containing three different MC-38-specific neo-antigens (Adpgk, Reps1 [13] and Rpl18 [14]) and a TLR2/4 agonist. VSV-GP-Mad46 was also generated de novo and contained the same antigen stretch as KISIMA-Mad46. In short, the peptide sequence of KISIMA-Mad46 was back-translated, codon-optimized for mus musculus and cloned into position 5 of the VSV-GP genome. All viruses were rescued and produced as described in [12].

### 2.5. Vaccination

Mice were either vaccinated subcutaneously (s.c.) with 2 nm KISIMA peptide or intravenously (i.v.) with 10^7^ TCID_50_ VSV-GP. Vaccination schedules for the respective tumor models were performed as described [12].

### 2.6. Tissue Processing

#### 2.6.1. Spleen

Spleens were dissected, mashed through a 70 μm cell strainer and washed with washing media (DMEM supplemented with 10% FCS, 2 mM Glutamine and Pen/Strep). Mononuclear cells were enriched using a density gradient medium (Lymphoprep™, Stemcell Technologies, Vancouver, BC, Canada). For the ELISpot TCR avidity assay and intracellular cytokine staining, splenocytes were eluted in CTL media (DMEM high glucose GlutaMAX, 6%FCS, 10 mM HEPES, 20 μM β-Mercaptoethanol, 0.66 mM L-Arginine, 0.24 mM L-Asparagine, 2 mM L-Glutamine, Pen/Strep).

#### 2.6.2. Lymph Nodes

Inguinal LNs were cut into 1mm pieces and digested with 1 mL DMEM containing 10 μg/mL DNAse and 1 mg/mL Collagenase D for 30 min at 37 °C. Cells were washed and eluted in flow cytometry cell sorter (FACS) buffer (PBS containing 2% FSC and 0.05% Na-Azide) or CTL media depending on the assay.

#### 2.6.3. Tumor Tissue

Tumors were harvested and weighed, and a maximum of 0.8 g of tumor tissue was used for digestion in mouse tumor dissociation buffer (Miltenyi Biotec, Bergisch Gladbach, Germany) utilizing the GentleMax dissociator according to the manufacturer’s specifications.

#### 2.6.4. Blood

Tail vein blood was collected in a tube containing 20 μL Heparin and enriched via density gradient centrifugation with Lymphoprep™.

#### 2.6.5. Bone Marrow

Bone marrow-dendritic cells (BM-DCs) were isolated from the femur and tibia, cultured, and activated (LPS) as previously described [15]. For intratumoral CD8+ T-cell polyfunctionality assays, BM-DCs were loaded with the corresponding peptide for 1 h, washed and co-cultured at a concentration of 0.2 × 10^5^ BM-DCs per 2 × 10^6^ TILs for 6 h.

### 2.7. FACS Analysis

Single-cell suspensions were prepared as described above, and antigen-specific CD8+ T-cell detection, surface FACS staining, intra-tumoral polyfunctionality assay and Granzyme B detection (for antibodies and tetramers see Tables S2 and S3) were performed as previously published [12]. The absolute number of tumor-infiltrating immune cells was quantified using CountBright™ absolute counting beads (Thermo Fisher Scientific, Waltham, MA, USA). Briefly, 100 µ L of the total tumor extract were stained for the CD45 marker, and the same number of beads was acquired by flow cytometry in order to back calculate the total number of cells recovered from the initial mass of tumor. A subsequent measurement was conducted on Attune (Thermo Fisher) and analyzed with Kaluza (Beckman Coulter, Brea, CA, USA) (Figures S4A,B, S5A,B and S6A).

### 2.8. TCR Avidity Assay

Either 0.5 × 10^6^ or 1 × 10^6^ splenocytes were plated in an IFN-γ detection antibody-coated plate (Diaclone, Besancon, France) and incubated with log_10_-fold dilutions of the indicated peptide (10 ug/mL to 10^−8^ ug/mL) at 37 °C, with 8%CO_2_. Additionally, each sample was incubated without peptides as controls to subtract the background signal. After 24 h IFN-γ spots were detected following the manufacturer’s protocol (Diaclone murine IFN-γ ELISpot Kit) on an AID Reader (AID GmbH, Bavaria, Germany), this was followed by analysis with AID ELISpot Reader Version 7.0 (AID GmbH). Wells were normalized to the highest peptide concentration well (100%), and significance for LogEC50 was calculated using non-linear regression with variable slope (four parameters) and an extra-sum-of-square F-test.

### 2.9. Cytokine Quantification

Tumor tissue was homogenized with 1 × RIPA buffer (0.4 mL per 0.1 g of tumor tissue) containing protease inhibitor (Roche, Basel, Switzerland) in the GentleMax dissociator (Miltenyi Biotec, Bergisch Gladbach, Germany). Following several centrifugations (14,000× *g*, 4 °C, 10 min), clear protein lysates were aliquoted for further analysis. The protein concentration was determined by the Pierce™ BCA Protein Assay Kit (Thermo Scientific, Waltham, MA, USA). LN cell suspensions were plated at 2 × 10^5^ cells/well and either incubated with CTL media or co-stimulated with anti-CD3 (0.5 ug/mL) and anti-CD28 (1 ug/mL) (Table S1). After 24 h, cells were removed by centrifugation, and the supernatant was aliquoted. Tumor lysates and LN supernatants were analyzed with a custom-made Luminex panel (Thermo Fisher Scientific, Waltham, MA, USA) for CD27, IFN-alpha, IFN-gamma, IL-1-β, IL-10, IL-12p70, IL-15, IL-17A, IL-2, IL-4, IL-6, IP-10/CXCL10, MCP-1/CCL2, MCP-3/CCL7, MIG/CXCL9 and RANTES/CCL5 (Figure S7A,B). Cytokine concentrations were measured with the MAGPIX™ System (Luminex) and Luminex xPONENT software 4.2.

### 2.10. Data Analysis

FACS data were analyzed with the Kaluza software (Beckman Coulter, Brea, California, USA), while Luminex data were evaluated with Procarta Plex Analyst version 1.0 (Luminex Corporation, Austin, TX, USA). Statistical analyses were performed using GraphPad Prism 9 (San Diego, CA, USA) and were considered significant if *p*-value <0.05. Statistical tests include a one-way or two-way ANOVA following Tukey’s multiple comparison test; two-tailed unpaired Mann-Whitney test; simple or non-linear regression with variable slope (four-parameters) with an extra-sum-of-square F-test; and a Log-rank (Mantel-Cox test). Treatment schemes were created with BioRender.com.

## 3. Results

### 3.1. Heterologous Prime-Boost Increases the Infiltration of Cross-Presenting cDC1s in Tumor and Tumor-dLNs

It was previously shown that the heterologous combination of the protein KISIMA (K) vaccine together with viral vector VSV-GP (V), both expressing tumor-associated antigens, induced long-term memory formation, increased T-cell polyfunctionality and improved treatment efficacy compared to their homologous counterparts [12].

**Figure 1:**
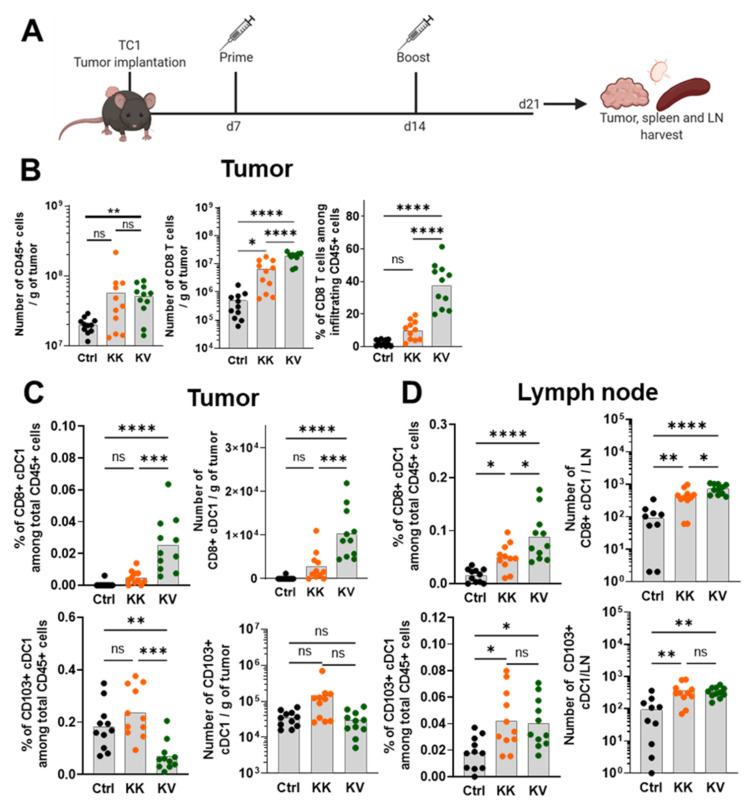
KV treatment induces cross-presenting cDC1s along with an increase in tumor-infiltrating CD45+ and CD8+ cells. (**A**) Treatment schedule of either heterologous prime (KISIMA-Mad25 peptide s.c.) and boost (VSV-GP-HPV i.v.) or homologous (twice KISIMA-Mad25 s.c.) vaccination before organ harvest for immune cell analysis of tumor, spleen and lymph nodes via FACS, Luminex and ELISpot. (**B**) From left to right: Number of CD45+ cells per gram tumor, number of CD8+ T cells per g tumor and % of CD8+ T cells among CD45+ cells derived from TC-1 tumor-bearing female C57BL/6J mice. (**C**) Frequencies (left) or absolute numbers (right) of either tissue-resident CD8+ cDC1s (upper graphs) or migratory CD103+ cDC1s (lower graphs) infiltrating TC-1 tumors. (**D**) Frequencies (left) or absolute numbers (right) of either tissue-resident CD8+ cDC1s (upper graphs) or migratory CD103+ cDC1s (lower graphs) derived from inguinal lymph nodes in TC-1 tumor-bearing C57BL/6J mice. Data in (**B**–**D**) are shown as means (grey bar) and derived from two independent experiments. * *p* < 0.05, ** *p* < 0.01, *** *p* < 0.001, **** *p* < 0.0001 One-Way ANOVA followed by Tukey’s multiple comparison test in (**B**–**D**) except for the number of CD45+ cells per gram tumor (**B**) where a two-tailed non-parametric Mann–Whitney test was performed (wide KK distribution) (** *p* < 0.01).

To further assess the immunological changes induced by the heterologous treatment combination, TC-1 tumor-bearing mice were primed s.c. with KISIMA-Mad25 (HPV-E7 epitope) followed by an i.v. immunization boost with VSV-GP-HPV (HPV-E2-E6-E7 epitopes) (Figure 1A). As previously shown [12], tumor size reduction in mice treated with the KV regimen began between days 5–7 post-boost (Figure S1A). Therefore, TC-1 tumors, tumor-draining LNs (-dLNs) and spleens were harvested at day 6 post-VSV-GP-HPV boost. Immune cell populations and their phenotype were analyzed in-depth. KV treatment significantly increased the overall infiltration of CD45+ leukocytes into the tumor compared to the controls, whereas the increase in the homologous KK treatment group was not statistically significant. We have previously shown that KV vaccination attracted the highest proportion of CD8+ T cells, CD4+ T-helper cells and increased the TAM-1/TAM-2 ratio, thereby favorably remodeling the TME [12]. In this study, we confirmed an elevated CD8+ T-cell tumor infiltration both in frequency and numbers in the KV-treated mice when compared to KK-treated and control mice (Figure 1B). The KK treatment group also displayed a moderate increase in CD8+ T cells in numbers but not frequency when compared to controls (Figure 1B). Furthermore, a modest augmentation of NK cell numbers was detected in both KV and KK-treated mice in comparison to controls (Figure S1B).

Among tumor-infiltrating CD45+ leukocytes, the percentage and the absolute number of CD8+/tissue-resident conventional DCs (cDC1s) were significantly increased in KV-treated mice when compared to KK-treated and control mice (Figure 1C). Furthermore, the frequency of CD103+/migratory cDC1s was reduced in the KV group; however, this was not translated into decreased absolute numbers (Figure 1C).

Migratory cDC1s take up tumor antigens, migrate to tumor-dLNs and cross-present antigens to CD8+ T cells [16]. Consequently, the presence of cDC1s subsets in tumor-dLNs was examined, and CD8+ tissue-resident cDC1s frequency and number were significantly elevated in the KK group compared to controls and further increased in the KV group (Figure 1D). Similarly, a significant increase in CD103+ migratory cDC1 was detected in both vaccinated groups compared to controls.

In addition to cross-presenting cDC1s, the presence of other DC subtypes with distinct properties was also monitored in the tumor and tumor-dLNs. Amongst these, cDC2s, crucial for the activation of CD4+ T-helper cells in tumor-dLNs [17], were found to be increased in tumor-dLNs and tumors of KK and KV-vaccinated mice compared to untreated controls (Figure S1C,D). Furthermore, it was observed that plasmacytoid DCs (pDCs), the main type-I IFN producers upon viral infections with the ability to display antigen-presenting function [18,19], the most profound changes were observed in tumor-dLNs with KV-treated animals showing the highest frequency compared to KK and controls, but in contrast, KV and KK exhibited equally higher total numbers compared to controls (Figure S1D). Lastly, monocytic DCs (moDCs) were assessed because their role in cancer immunotherapy remains context-dependent and extent of inflammation-dependent and can range from the activation of CD8+ T cells and the induction of a Th1-biased CD4+ T-helper cell responses to their promotion of Treg cell proliferation [20]. As depicted in Figure S1D, moDCs were significantly higher in tumor-dLNs in KV-treated compared to KK and controls.

### 3.2. KV Induces a Th1-Biased T-Helper Cell Profile

**Figure 2:**
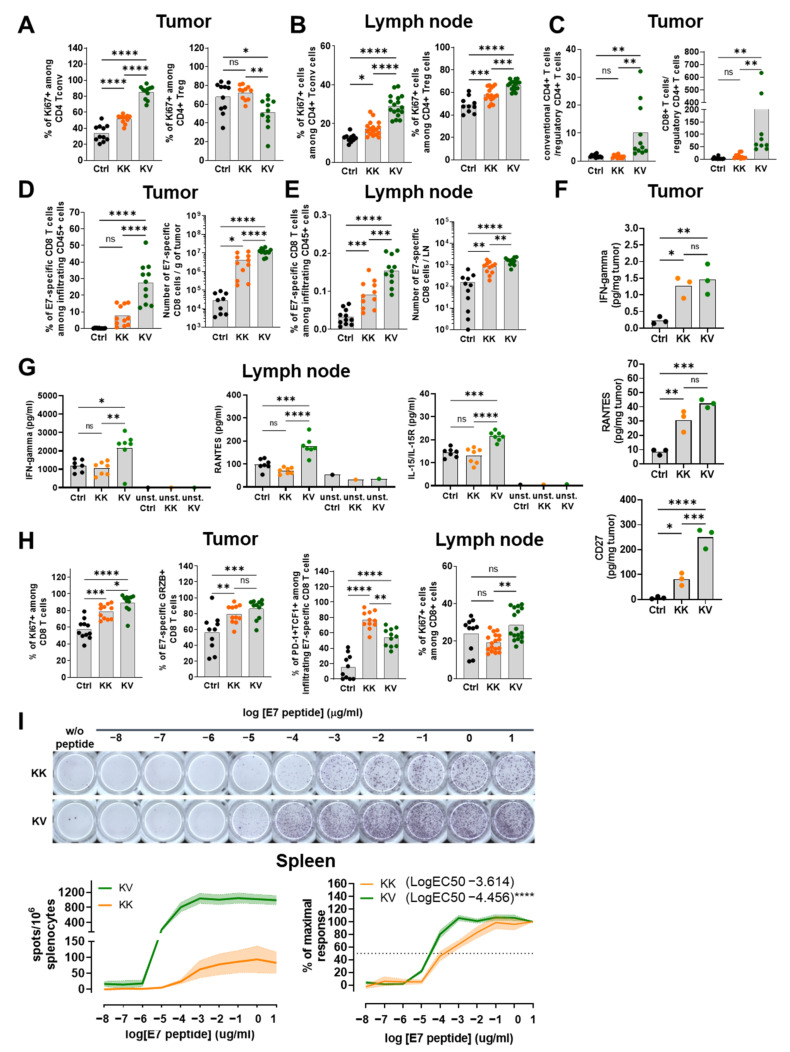
Improved CD4+ and CD8+ T-cell characteristics in tumor and tumor-dLNs and TCR avidity in the spleen of heterologous-treated TC-1 tumor-bearing C57BL/6 mice. (**A**) % of intratumoral Ki67+ cells among FoxP3- CD4+ Tconv cells (Tconv-right side) or % of intratumoral Ki67+ cells among FoxP3+ CD25+ CD4+ regulatory T cells (Treg-right side) derived from TC-1 tumor-bearing mice on day 21 post-tumor implantation. (**B**) % of inguinal, intranodal Ki67+ cells among FoxP3- CD4+ Tconv cells (Tconv-left side) or % of inguinal, intranodal Ki67+ cells among FoxP3+ CD25+ CD4+ regulatory T cells (Treg-right side) derived from TC-1 tumor-bearing mice on day 21 post-tumor implantation. (**C**) Ratio of intratumoral Tconv to Treg (left side) or intratumoral CD8+ T cells to Treg derived from TC-1 tumor-bearing mice on day 21 post-tumor implantation. (**D**) % (left) or number per gram tumor (right) of antigen-specific (E7) CD8+ T cells derived from TC-1 tumor-bearing mice on day 21 post-tumor implantation. (**E**) % (left) or number (right) per inguinal LN of antigen-specific (E7) CD8+ T cells derived from TC-1 tumor-bearing mice on day 21 post-tumor implantation. (**F**) Cytokine (IFN-γ, RANTES, CD27) concentrations in pg/mL derived from tumor lysates which were harvested on day 21 post-tumor implantation and measured by Luminex (Figure S7A). (**G**) Cytokine (IFN-γ, RANTES, IL-15/IL-15R) concentrations in pg/mL derived from lymph node cell suspension supernatants 24 h after re-stimulation with α-CD3/α-CD28 beads or respective controls, which were harvested on day 21 post-tumor implantation and measured by Luminex (Figure S7B). (**H**) From left to right: % of Ki67+ cells among tumor-infiltrating CD8+ T cells, % of Granzyme B+ cells among E7+ CD8+ tumor-infiltrating T-cells, % of PD-1+ Tcf-1+ cells among E7+ CD8+ tumor-infiltrating T-cells, and % of Ki67+ cells among intranodal CD8+ T cells on day 21 post-TC-1 tumor implantation. (**I**) Representative IFN-γ spot images of KK or KV derived splenocytes harvested on day 21 post-TC-1 tumor implantation and incubated for 24 h with varying concentrations of E7 peptide (upper left). Dose–response curve of IFN-γ spots per 106 splenocytes incubated with log-fold dilutions of E7 peptide (lower left). TCR avidity by IFN-γ spots (% to maximal response) determined by differences in LogEC50. Data in (**A**–**E**,**H**,**I**) are shown as means (grey bar) and derived from two independent experiments. Data in (**F**,**G**) derive from one experiment. * *p* < 0.05, ** *p* < 0.01, *** *p* < 0.001, **** *p* < 0.0001 One-Way ANOVA followed by Tukey’s multiple comparison test. Data in (**F**) derives from two independent experiments with n = 10 for KK and n = 11 for KV. **** *p* < 0.0001 extra-sum-of-square F-test.

Another important factor for developing superior CD8+ T-cell polyfunctionality is the presence of appropriate CD4+ T cell help [17,21]. To analyze CD4+ T-cell polarization, T helper-specific intra-cellular transcription factor staining in tumor and tumor-dLNs was performed. KV heterologous treatment significantly increased Th1 CD4+ T cells (% and numbers) in the tumor, while other T-helper subtypes (Th2, Th17, Tregs) were reduced in frequencies, but not in absolute numbers. Similar results, consisting of a Th1-dominant T-helper profile, were obtained in tumor-dLNs (Table 1 and Figure S2H–G). Next, we assessed whether the CD4+ T-cell compartment in KV-treated animals exhibited a better proliferation capacity via the upregulation of the Ki67 marker. Conventional CD4+ T cells (Tconv) from vaccinated mice defined as non-Treg cells demonstrated a significantly greater proliferation capacity in tumor and dLNs (Figure 2A,B). In contrast, Treg cells expressed less Ki67 in the tumor but more in the tumor-dLNs of KV-treated mice (Figure 2A,B). In the context of tumor therapy, an inverse ratio of intratumoral effector CD8+/CD4+ T cells to immune-suppressive Tregs can be a prognostic factor for better overall survival [22]. Importantly, an inverse CD4+ Tconv cells-to-CD4+ Tregs ratio, as well as a higher CD8+ T cell-to-CD4+ Tregs ratio, was observed after KV treatment in the tumor but not in tumor-dLNs (Figure 2C and Figure S1E).

### 3.3. KV Elicited CD8+ T Cells Exhibit Superior Treatment Efficacy Not Only via Increased Quantities but Also Improved Quality

Previous findings regarding CD8+ T-cell polyfunctionality [12] were confirmed with enhanced E7-specific CD8+ T-cell infiltration into the tumor along with enhanced E7-specific CD8+ T-cell polyfunctionality, defined by the simultaneous expression of IFN-γ, TNFα and the degranulation marker CD107α along with a less exhausted phenotype (CD8+ PD1+ Tim3+) (Figure 2D and Figure S2A). In addition, KK treatment increased the frequency and number of E7-specific CD8+ T cells in tumor-dLNs, and this effect was further enhanced in the KV-treated group (Figure 2E). These findings were accompanied by cytokine and chemokine release measurements that revealed elevated levels of IFN-g and RANTES (CCL5) in both the tumor and tumor-dLNs with the highest values found in KV followed by KK and control groups (Figure 2F,G). Additionally, elevated levels of IL-15/IL-15R were found in tumor-dLNs, possibly indicating the formation of long-term memory [23]. Moreover, the expression of the co-stimulatory receptor CD27 has been shown to promote CTL effector and memory formation [24] and to increase the survival of clonally expanded CD8+ T cells [25]. Interestingly, increased levels of CD27 in the tumor were detected in KV-treated mice when compared to KK-treated mice, the latter displaying higher CD27 levels compared to controls (Figure 2F).

Furthermore, CD8+ T cells from KV-treated mice exhibited increased proliferation compared to KK and controls (Figure 2H). With regards to the intra-tumoral CD8+ T-cell characterization, a higher frequency of granzyme B+ E7-specific CD8+ T cells was found in the heterologous KV group. An increased stemness of E7-specific CD8+ T cells (PD-1+Tcf-1+) was also observed in both treatment groups (Figure 2H). Although KK displayed slightly higher frequencies than the KV group, the increased frequency and number of intra-tumoral E7-specific CD8 T cells in KV-treated mice compensate for this difference.

The afunctional TCR avidity assay evaluates the antigen sensitivity of a CD8+ T-cell pool by detecting the formation of IFN-g spots in the presence of decreasing antigenic peptide concentrations. Splenic CD8+ T cells from KV-treated mice showed significantly increased functional TCR avidity compared to the KK group, characterized by an almost one-log increase in LogEC50 (Figure 2I).

In conclusion, these data highlighted the superior anti-tumoral response induced by a heterologous KV vaccination, not only affecting antigen-specific CD8+ T-cell infiltration but also improving their functionality, all the while positively impacting DCs recruitment and CD4+ T-cells polarization towards anti-tumoral Th1 phenotype.

### 3.4. KVK-Mad46 Prolongs Overall Survival and Induces Long-Term Remission in the MC-38 Tumor Model

Homologous KISIMA and heterologous KVK combinations with two MC-38 neo-antigen-specific CD8+ T-cell epitopes (Adpgk and Reps1) have been previously tested [12]. To further improve the efficacy of this multi-epitope vaccine, a recently discovered neo-antigen (Rpl18), the main driver for endogenous CD8+ T-cell response in the MC-38 colon adenocarcinoma [14], was included in a newly designed KISIMA construct (KISIMA-Mad46 comprising Adpgk-, Reps1- and Rpl18-derived epitopes) as well as into the VSV-GP backbone (VSV-GP-Mad46).

**Figure 3:**
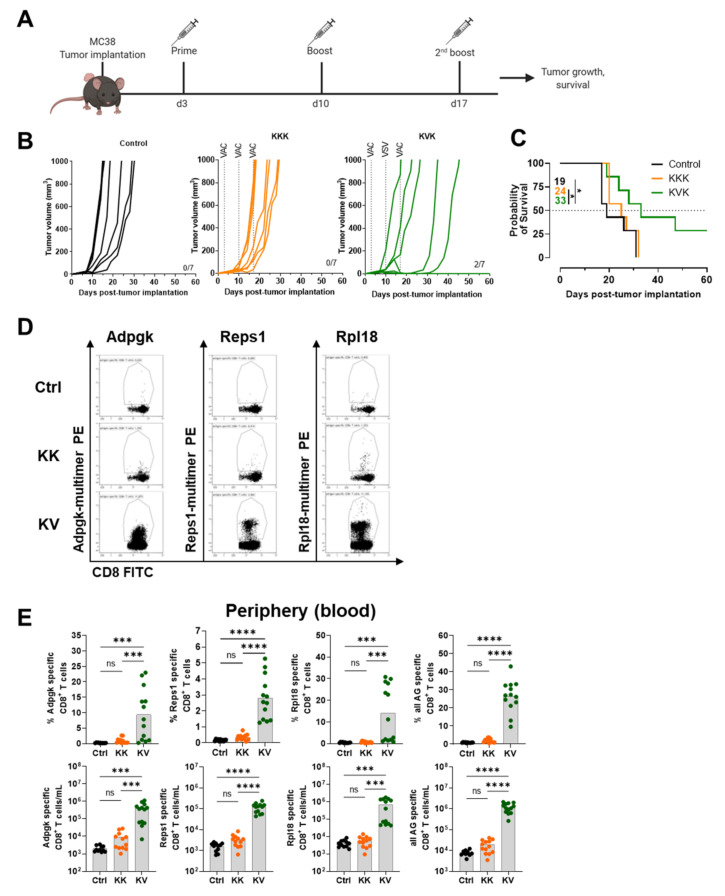
Improved efficacy and increase in multiple antigen-specific CD8+ T cells in the periphery of heterologous prime-boost-treated MC-38 tumor-bearing C57BL/6 mice. (**A**) Treatment schedule of prime (KISIMA-Mad46 s.c.), boost (VSV-GP-Mad46 i.v. or KISIMA-Mad46 s.c.) an^d^ 2nd boost (KISIMA-Mad46 s.c.) vaccination after MC-38 tumor implantation in female C57BL/6 mice for monitoring tumor-growth and prolonged overall survival. (**B**) Individual tumor-growth curves in mm³ post-tumor implantation for untreated controls, KKK or KVK-treated female C57BL/6 mice. (**C**) Kaplan–Meier survival graphs of MC-38 tumor-bearing female C57BL/6 mice (n = 7 per group). (**D**) Representative FACS dot plots of blood-derived Adpgk, Reps1 and Rpl18-specific CD8+ T cells. From left to right: Control, KK or KV treatment group is shown on day 18 post-MC-38 tumor implantatio (**E**) From left to right: % (upper part) or absolute numbers (lower part) of blood-derived Adpgk-, Reps1-, Rpl18- or all three (sum) antigen-specific CD8+ T cells sampled on day 16 or 17 post-MC-38 tumor implantation. Data in (**D**,**E**) are shown as means (grey bar) and derived from two independent experiments. * *p* < 0.05, *** *p* < 0.001, **** *p* < 0.0001 One-Way ANOVA followed by Tukey’s multiple comparison test. Data shown in (**B**,**C**) are derived from one experiment (n = 7). * *p* < 0.05 Mantel-Cox Log-rank test.

To evaluate the efficacy of our new multi-epitope vaccine in a homologous versus heterologous prime-boost regimen, we s.c. implanted MC-38 tumors into mice. These mice were vaccinated with KISIMA-Mad46 after three days and boosted twice in a 7-day interval with either KISIMA-Mad46 or VSV-GP-Mad46 (Figure 3A). We showed that KVK treatment substantially prolonged the overall survival of mice compared to the KKK treatment and controls. In addition, the KVK group exclusively led to complete tumor regression in two out of seven animals (Figure 3B).

Monitoring the peripheral antigen-specific CD8+ T-cell response seven days after the first boost immunization unveiled the highest frequency of Adpgk-, Reps1- and Rpl18-specific CD8+ T cells in the heterologous treatment group (Figure 3D,E). Interestingly, no dominant epitope was consistently identified as the response to the three antigens differed from animal to animal despite the Reps1-specific response being the weakest one (Figure 3E). Together, these data showed that combining all three antigens (all Ag) resulted in significantly elevated frequencies and numbers (Figure 3E).

### 3.5. KV Increases the Migration of Antigen-Specific CD8+ T Cells into the Tumor and Tumor-dLNs of MC-38 Tumor-Bearing Mice

**Figure 4:**
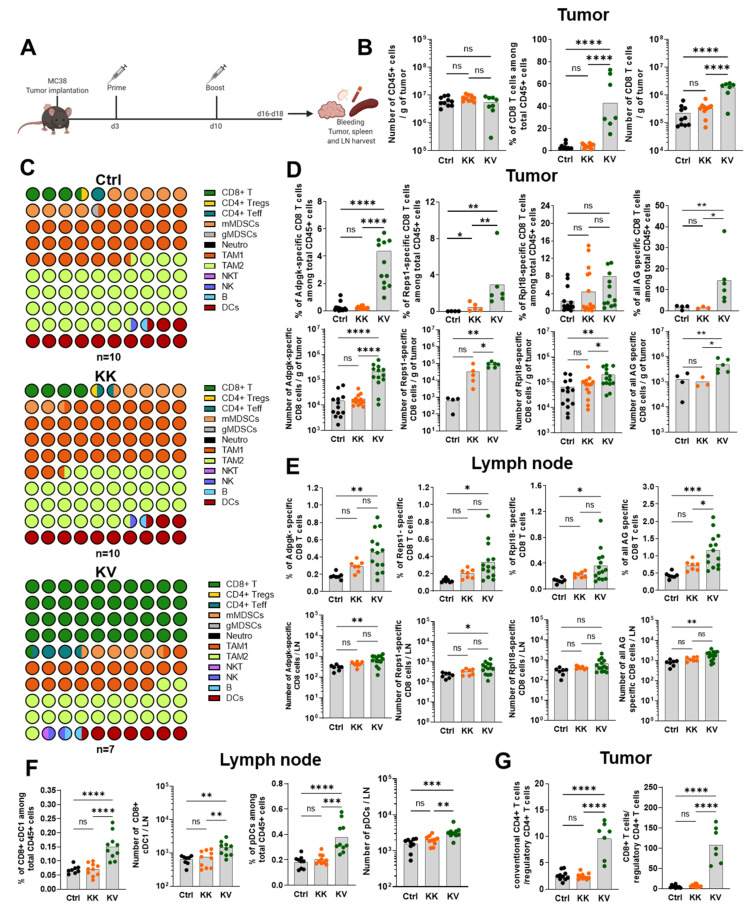
Increased antigen-specific CD8+ T cell in tumor and tumor dLNs and elevated intranodal CD8+ cDC1s and pDCs of heterologous prime-boost-treated MC-38 tumor-bearing C57BL/6 mice. (**A**) Treatment schedule of either heterologous prime (KISIMA-Mad46 peptide s.c.) and boost (VSV-GP-Mad46 i.v.) or homologous (twice KISIMA-Mad46 s.c.) vaccination before organ harvest for immune cell analysis via FACS and ELISpot. (**B**) The number of CD45+ cells per gram tumor (left), % of CD8+ T cells among tumor-infiltrating CD45+ cells (middle) and the number of CD8+ T cells per gram tumor (right) at day 18 post-MC-38 tumor implantation. (**C**) In-depth TIL characterization by flow cytometry on day 18 post-MC-38 tumor implantation in untreated controls (Ctrl, n = 10), homologous KISIMA-Mad46 (KK, n = 10) or heterologous KISIMA-Mad46/VSV-GP-Mad46 (KV, n = 7) treated animals. Various immune cells were measured, as indicated in the legend. For mean values ±SEM, see Table S1. (**D**) From left to right: % (upper part) or absolute numbers (lower part) of tumor-infiltrating Adpgk-, Reps1-, Rpl18- or all three (sum) antigen-specific CD8+ T cells at day 18 post-MC-38 tumor implantation. (**E**) From left to right: % (upper part) or absolute numbers (lower part) of intranodal Adpgk-, Reps1-, Rpl18- or all three (sum) antigen-specific CD8+ T cells at day 18 post-MC-38 tumor implantation. (**F**) Frequencies or absolute numbers of intranodal tissue-resident CD8+ cDC1s (left) or migratory plasmacytoid DCs (pDCs) (right) at day 18 post-MC-38 tumor implantation. (**G**) Ratio of intratumoral Tconv to Treg (left) or intratumoral CD8+ T cells to Treg (right) derived from MC-38 tumor-bearing mice on day 18 post-tumor implantation. Data in (**B**,**C**,**E**–**G**) are shown as mean (grey bar) and derive from at least two independent experiments. * *p* < 0.05, ** *p* < 0.01, *** *p* < 0.001, **** *p* < 0.0001 One-Way ANOVA followed by Tukey’s multiple comparison test. Adpgk- and Rpl18-specific CD8+ T-cell analysis from (**D**) is derived from three independent experiments, * *p* < 0.05, ** *p* < 0.01, *** *p* < 0.001, **** *p* < 0.0001 One-Way ANOVA followed by Tukey’s multiple comparison test. Reps1 and all AG-specific CD8+ T cells from (**D**) derive from one experiment, * *p* < 0.05, ** *p* < 0.01, *** *p* < 0.001, **** *p* < 0.0001 One-Way ANOVA followed by Tukey’s multiple comparison test or two-tailed unpaired Mann–Whitney test.

Next, we investigated the changes of immune cell infiltrations induced by the KV treatment. MC-38 tumors, tumor-dLNs and spleens were harvested from mice post-boost (Figure 4A) during the remission phase but before complete tumors regression in the KV group (Figure S2B). An in-depth immune cell characterization was performed. Contrarily to the TC-1, the MC-38 tumor is considered a “hot, inflamed” tumor with high CD11b+ leukocyte infiltration [26]. As such, no increase was observed in CD45+ infiltrating leukocytes between control, KK- or KV-treated groups (Figure 4B). However, KK treatment created a beneficial shift of the TAM1/TAM2 ratio towards TAM1, which is pro-inflammatory TAM1 (Figure 4C).

Subsequently, the antigen-specificity of infiltrating CD8+ T cells was characterized. Following the pattern observed in the periphery, KV-treated animals exhibited the highest frequency and number of Adpgk- and Reps1-specific CD8+ T cells. As Rpl18 is known to be the main endogenous CD8+ T-cell clone in the MC-38 model, approximately 3% of Rpl18-specific CD8+ T cells were observed in controls, and in contrast to the periphery, their frequency was not increased by either KK or KV treatment. Furthermore, a statistically significant increase in the total number of Rpl18-specific CD8+ T cells was observed in KV-treated mice when compared to KK and controls. Similar to as in the blood, the frequency and number of tumor-infiltrating antigen-specific CD8+ T cells were further increased when the sum of Adpgk-, Reps1- and Rpl18-specific CD8+ T cells was considered. With regards to tumor-dLNs, KV vaccination significantly increased Adpgk-, Reps1- and Rpl18-specific CD8+ T cells compared to controls (Figure 4E).

### 3.6. KV Reduces Tumor-Infiltrating CD4+ Tregs Whilst Increasing CD8+ cDC1s and pDCs in Tumor-dLNs

Following KV treatment, there was no difference in tissue-resident cDC1s, and a decrease in migratory cDC1s in frequencies and numbers was observed in the MC-38 model (Figure S2D). The intratumoral frequencies and numbers of cDC2s, moDCs and pDCs did not differ between groups but were generally higher than in the TC-1 tumor model, potentially due to the high natural influx of CD11b+ cells into MC-38 tumors (Figure S2E). Additionally, increased frequencies and numbers of tissue-resident CD8+ cDC1s and pDCs were detected in tumor-dLNs of KV compared to KK and control groups (Figure 4F). In contrast, no changes were observed between groups in intranodal migratory CD103+ cDC1s, cDC2s or moDCs (Figure S2F).

Moreover, while the T-helper subtype composition did not differ in the tumor or in tumor-dLNs between treatment groups, the number and frequency of intratumoral CD4+ Tregs were significantly reduced in the KV group (Figure S3A,B). Consequently, the ratio of intratumoral CD4+ Tconv cells to CD4+ Tregs as well as the ratio of intratumoral CD8+ T cells to CD4+ Tregs were considerably increased in KV-treated mice compared to KK-treated and controls (Figure 4G).

### 3.7. Polyfunctionality, Exhaustion Status and TCR Avidity Differ between Adpgk-, Rpl18- and Reps1-Specific CD8+ T Cells

**Figure 5:**
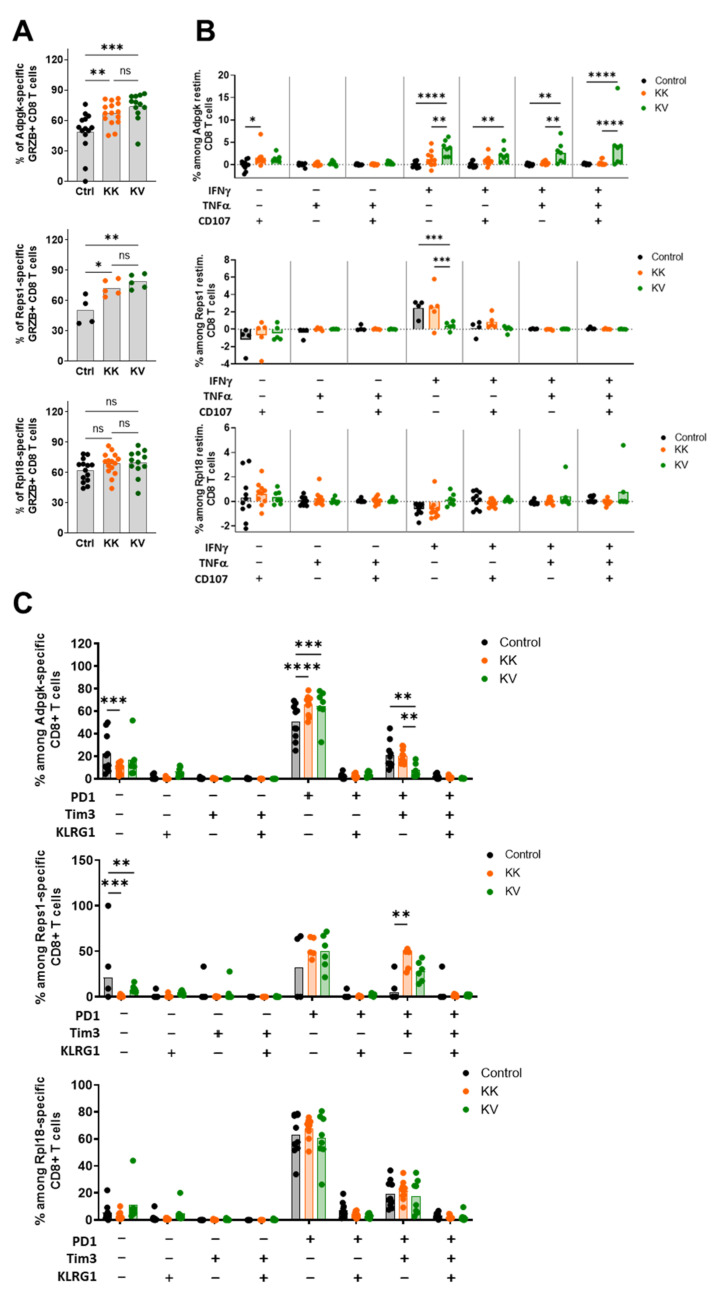
Different functional and exhaustion status among antigen-specific tumor-infiltrating CD8+ T cells in MC-38 tumor-bearing C57BL/6 mice. (**A**) Intracellular Granzyme B positive CD8+ T cells were assessed among tetramer-positive (from top to bottom: Adpgk, Reps1, Rpl18) intratumoral CD8+ T cells. Tumors were harvested on day 18 post-MC-38 tumor implantation. (**B**) Tumor-infiltrating CD8+ T cells were stimulated for 6 h with either Adpgk (upper graph), Reps1 (middle) or Rpl18 (lower graph) peptide and stained for IFN-γ, TNF-α and CD107α. Tumors were harvested on day 18 post-MC-38 tumor implantation. (**C**) The exhaustion status was examined on tumor-infiltrating tetramer-positive (from top to bottom: Adpgk, Reps1, Rpl18) CD8+ T cells in regard to expression of PD-1, Tim-3 and the effector marker KLRG1. Tumors were harvested on day 18 post-MC-38 tumor implantation. Data in (**A**–**C**) are shown as means (filled bar) and derived from at least two independent experiments for Adpgk and Rpl18 analysis and one experiment for Reps1. * *p* < 0.05, ** *p* < 0.01, *** *p* < 0.001, **** *p* < 0.0001. One-Way ANOVA followed by Tukey’s multiple comparison test.

Then, we analyzed the characteristics and functionality of the different antigen-specific CD8+ T cells. An augmentation of Adpgk- and Reps1- but not Rpl18-specific CD8+ T cells expressing Granzyme B in KK- and KV-treated mice compared to controls (Figure 5A). In addition, polyfunctional Adpgk but not Reps1 nor Rpl18-specific tumor-infiltrating CD8+ T cells were observed in the KV group (Figure 5B). This directly correlated with a reduction in exhausted PD-1+, Tim-3+ Adpgk-specific CD8+ T cells among KV-treated mice, while exhaustion markers on Rpl18- and Reps1-specific CD8+ T cells remained similar between all three groups (Figure 5C).

**Figure 6:**
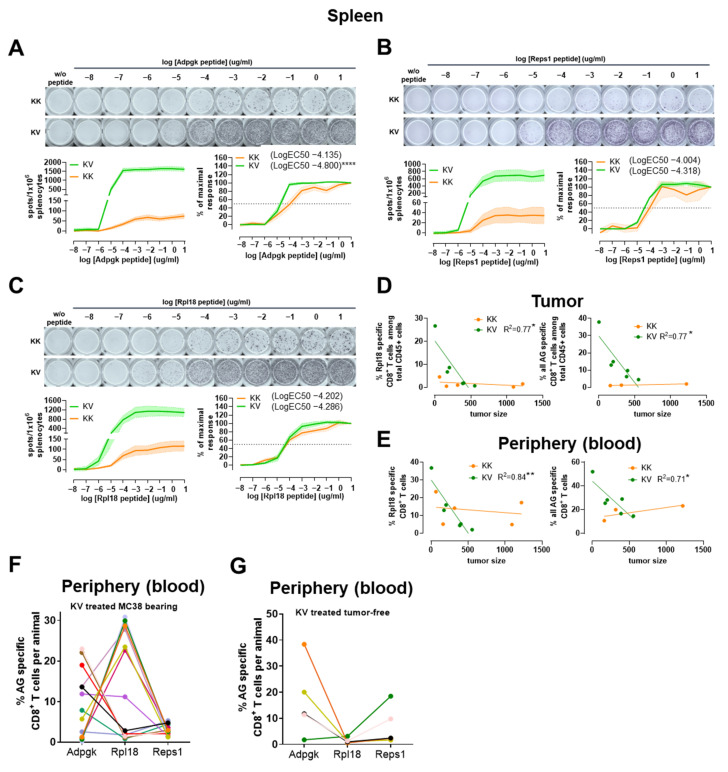
TCR avidity: the correlation of antigen-specific CD8+ T cells to tumor size and antigen competition in MC-38 tumor-bearing or non-tumor-bearing C57BL/6 mice. (**A**) Representative IFN-γ spot images of KK or KV derived splenocytes harvested on day 18 post-MC-38 tumor implantation and incubated for 24 h with varying concentrations of Adpgk peptide respectively (top). Dose–response curve of IFN-γ spots per 106 splenocytes incubated with log-fold dilutions of Adpgk peptide (left). TCR avidity by IFN-γ spots (% to maximal response) determined by differences in LogEC50 (right). (**B**) Representative IFN-γ spot images of KK or KV derived splenocytes harvested on day 18 post-MC-38 tumor implantation and incubated for 24 h with varying concentrations of Reps1 peptide, respectively (top). Dose–response curve of IFN-γ spots per 106 splenocytes incubated with log-fold dilutions of Reps1 peptide (left). TCR avidity by IFN-γ spots (% to maximal response) determined by differences in LogEC50 (right). (**C**) Representative IFN-γ spot images of KK or KV derived splenocytes harvested on day 18 post-MC-38 tumor implantation and incubated for 24 h with varying concentrations of Rpl18 peptide, respectively (top). Dose–response curve of IFN-γ spots per 106 splenocytes incubated with log-fold dilutions of Rpl18 peptide (left). TCR avidity by IFN-γ spots (% to maximal response) determined by differences in LogEC50 (right). (**D**) Correlation graphs from KV-treated mice of tumor-infiltrating % of Rpl18-specific (left) or all AG-specific (right) CD8+ T cells to tumor size in mm³ on day 18 post-MC-38 tumor implantation. (**E**) Correlation graphs from KV-treated mice of blood-derived % of Rpl18-specific (left) or all AG-specific (right) CD8+ T cells to tumor size in mm³ on day 18 post-MC-38 tumor implantation. (**F**) Comparison of antigen frequencies between blood-derived Adpgk-, Rpl18- and Reps1-specific CD8+ T-cell frequencies among individual animals from heterologous (KV)-treated MC-38 tumor-bearing animals on day 7 post-boost immunization. (**G**) Comparison of antigen frequencies between blood-derived Adpgk-, Rpl18- and Reps1-specific CD8+ T-cell frequencies among individual animals from heterologous (KV)-treated animals on day 7 post-boost immunization. Data in (**A**–**C**) derive from at least two independent experiments. Data in (**A**–**C**) shows n-numbers with n = 10 for KK and n = 10–17 for KV with **** *p* < 0.0001 from extra-sum-of-square F-test. Data in (**D**,**E**) are derived from one experiment, * *p* < 0.05, ** *p* < 0.01 are calculated with simple linear regression. Data in (**F**) are derived from two independent experiments, and data in (**G**) are derived from one experiment.

The functional TCR avidity of the different antigen specificities (Adpgk, Reps1, Rpl18) was assessed in splenocytes from MC-38 tumor-bearing mice. A significantly higher LogEC50 concentration in KV derived Adpgk-specific CD8+ T cells was detected compared to KK (Figure 6A). Similar to the results of polyfunctionality analysis, neither Reps1- nor Rpl18-specific CD8+ T cells demonstrated statistically significant differences in LogEC50 between the two treatment groups (Figure 6B,C).

Lastly, when the frequency of the different antigen specificities was correlated with the tumor volume, a statistically significant inverse correlation was observed between tumor-infiltrating Rpl18- or all Ag-specific tumor-infiltrating CD8+ T cells and tumor volume in KV-treated but not KK-treated mice (Figure 6D). Interestingly, the same correlations were observed for the frequencies of Rpl18- or all Ag-specific CD8+ T cells found in the periphery (Figure 6E).

### 3.8. The Presence of MC-38 Tumor Impacts the Relationship between Adpgk-, Rpl18- and Reps1-specific CD8+ T-Cell Frequencies

We next assessed the impact of the presence of an implanted tumor on different antigen-specific CD8+ T-cell frequencies. Despite no clear trend for dominance of one antigen-specificity, the frequency of Rpl18-specific CD8+ T cells was higher in MC-38 tumor-bearing compared to tumor-free animals. This indicated a strong impact of the MC-38 tumor as a source of Rpl18 antigen, which was further boosted by heterologous vaccination (Figure 6F,G). In addition, a statistically significant inverse correlation between the frequency of Rpl18- and Adpgk-specific CD8+ T cells was observed, and this was further increased when the sum of Adpgk- and Reps1-specific was compared to the frequency of Rpl18-specific CD8+ T cells in the periphery of tumor-bearing animals (Figure S3C).

In summary, in the context of a multi-epitope vaccine, heterologous prime-boost treatment induced high frequencies of multiple antigen specificities in the periphery as well as in the tumor of heterologous-vaccinated animals.

## 4. Discussion

Heterologous prime-boost strategies in oncology have resulted in qualitatively broader immune responses compared to those elicited by unique modality vaccines [27,28,29,30]. We have recently demonstrated that combining the KISIMA vaccine with a VSV-GP viral vector expressing the same antigen stretch results in a potent heterologous prime-boost treatment in tumor-bearing mice [12]. In this setting, the improved efficacy of the vaccine is characterized by increased quantity and quality, limited exhaustion of CD8+ T-cell responses and a TME remodeled to acquire inflammatory properties allowing for the maintenance of anti-tumor CTL capacities. The pro-therapeutic effect observed after the heterologous prime-boost may also rely on the T-helper cell activation or on the T-cell priming either in the tumor-dLNs or at the tumor site by their specific DCs. While most studies have mainly focused on the characterization of CD8+ T cells, this study attempted to further investigate the other immune cells that are crucial to educating anti-tumoral CD8+ T cells, such as cross-presenting DCs and CD4+ T-helper cells [31].

In general, CD4+ Th1, infiltrating CD8+ CTLs and CD103+ cDC1s contribute to the main anti-tumor effects observed upon the induction of various immunomodulatory treatment regimens, including checkpoint inhibitors, oncolytic viruses, radiotherapy, chemotherapy, or adoptive T-cell therapy [32]. The KV regimen induces a Th1 dominant Thelper cell profile in the TC-1 tumor model, with higher proliferative capacity and reduced CD4+ Treg frequencies and proliferation. In contrast to TC-1, no Th1 dominance was shown in the “hot” MC-38 tumor model, characterized by high myeloid cell infiltration [26]. This difference could depend on the lack of CD4-specific epitopes in the KISIMA-Mad46 vaccine, or alternatively on the absence of a TAM1 skewed TAM1/TAM2 ratio, as TAM1 polarize CD4+ T cells towards Th1 phenotype [33].

Tregs migrate naturally to sites of infections, but the balance with CD4+ and CD8+ T cells and cytokines determines the outcome of an immune response, ranging from pro-inflammatory to anti-inflammatory [34]. The beneficial CD4+/Treg and CD8+/Treg ratios, observed in tumor-dLNs and in the tumor after heterologous prime-boost in both tumor models, correspond to clinical data that correlates a higher ratio of these cell types with prolonged survival while associating a high prevalence of Tregs with a poor prognosis in several human cancers [35].

cDC1 was recently shown to prime CD4+ and CD8+ T cells and can be licensed by CD4+ T cells in the setting of tumor-derived antigens [36]. The KV regimen inducing cross-presenting CD8+ cDC1s in TC-1 tumor and in TC-1 and MC-38 tumor-dLNs is in line with published data, showing that CD8+ cDC1s are critical for CTL responses [37], and tumor-dLN cDC1s contribute to CD8+ T-cell priming. In contrast, in both tumor models, the CD103+/migratory DC population was reduced at the tumor site after KV treatment, suggesting their migration towards the tumor-dLN to present tumor antigens to naive T cells, as described within the literature [38]. Regarding the remaining DC populations, the favorable increased frequency of cDC2s observed in both TC-1 tumor and tumor-dLNs, known to contribute to CD4+ T-cell activation [39], matches the increased CD4+ T-cell proliferation observed after vaccination. Although contradictory roles have been attributed to pDCs for CTL responses against tumor-derived antigens, the pDCs influx in tumor-dLNs observed in both models upon vaccination support cross-priming capacity [40] and type-I IFN-driven CTL boosting [41]. This is consistent with upregulated type-I IFN levels previously observed in TC-1 tumor-bearing animals after KV vaccination [12]. Additional migratory DCs, such as moDCs, that differentiate from monocytes in peripheral tissues upon inflammation promote context-dependent differentiation of CD4+ T cells towards a Th1, Th2 or Th17 phenotype [20] and possess cross-presenting capacity [42]. In the present study, moDCs might positively correlate with the observed Th1 and CTL increases upon KV treatment in the TC-1 tumor-dLNs.

The KV combination turns a “cold” into a “hot” TC-1 tumor as shown by the high infiltration of CD45+ T cells, one-third being tumor-specific CD8+ T cells. Here, we report an increased high-avidity of tumor antigen-specific CD8+ T cells in the spleen from KV-treated animals, alongside increased Granzyme B expression and elevated proliferation, TNF-α and IFN-γ secretion. Taken together, this highlights an improved and enhanced CTL response. Interestingly, in the KV setting, the increased CD4+ T cells may play a role in increasing the TCR functional avidity of CD8+ T cells, as reported by Zhu et al. [43]. CD4+ T-cell help may be responsible for the increased CD8+ T-cell proliferation as it was shown for E7-specific CD4+ T-cell help [44]. It is now widely accepted that fully activated CD8+ T cells require the participation of CD4+ T-helper cells through cytokine production and/or costimulatory molecule/receptor interaction between antigen-presenting DCs and CD8+ T cells [17]. The elevated release of CD27 in the tumor supports a potential CD4+ T-cell help, as observed in the TC-1 model. KISIMA-HPV and VSV-GP-HPV vaccines both contain CD4 epitopes; however, an antigen-specific CD4+T-cell help was not shown in the present study and will be the purpose of future studies.

KISIMA-Mad46 and VSV-GP-Mad46 combination, both comprising of three neo-antigens, Adpgk (H-2Db), Reps1 (H-2Db) and Rpl18 (H-2Kb), allowed for the characterization of multiple antigens and their respective contribution to the CD8+ T-cell response in the MC-38 model. The heterologous multi-epitope vaccination not only induced antigen-specific CD8+ T-cell responses in the periphery but also in the tumor and tumor-dLNs to all three antigens, thereby lowering the risk of escape mutants due to loss of antigen expression/mutations [14].

In contrast to the TC-1 model, KV vaccination in the MC-38 model amplifies CD8+ T-cell numbers despite not increasing total CD45+ T cells. At the antigen-specific level, TCR avidity differences correlate with polyfunctionality and the exhaustion status of each antigen-specific CD8 T cell. Differences were observed between the epitopes, with Adpgk being more polyfunctional than Rpl18 and Reps1. This may be due to different amounts of antigen present in the tumor, where either high or sometimes low amounts of antigen lead to higher responsiveness [45], and/or due to exhaustion generated by prolonged antigen stimulation [46]. A recent study showed that two H-2Kb restricted neo-antigens with variable MHC molecule binding affinity drives functional anti-tumor immunity, but one becomes immunodominant when combined [47]. In our case, this competition should not take place on the antigen presentation level as Adpgk and Rpl18 are presented on different MHCs, H-2Db and H-2Kb, respectively [14]. The variability of all three neo-antigen-specific CD8+ T-cell frequencies between each animal could be a consequence of an endogenous presence of intra-tumoral Rpl18-specific CD8+ T cells [14]. Consequently, when priming with our multi-epitope vaccine, Adpgk- and Reps1-specific CD8+ T cells are primed from naive T cells, whereas pre-existing Rpl18-specific CTLs experience a boost immunization resulting in faster re-activation and superior clonal expansion [48], which together leads to the observed inverse correlation of Adpgk- to Rpl18-specific CTLs. In support of this hypothesis, we did not observe this competition in non-tumor-bearing animals, where Rpl18-specific CD8+ T-cell frequencies were decreased compared to tumor-bearing animals arguing that the tumor influences this competition phenomenon. Other factors that contribute to the heterogeneity of the three respective neo-antigen-specific CD8+ T-cell responses observed between non-tumor-bearing mice could be due to epigenetic influences [49] or differences in their microbiomes [50]. Eventually, the various repartition among the three neo-antigen-specific CD8+ T-cell responses combined with the viral boost leads to improved efficacy in all animals and confirms the superiority of the treatment with a multi-epitope vaccine, as previously reported in the MC-38 model [51]. However, additional improvement of a multi-epitope vaccine and in relation to the MC-38 tumor model might be achieved by including tumor/MC-38 antigen-specific or bystander CD4+ T-helper cell epitopes in the future.

As CD4+ T-cell help and cross-presenting cDC1s enhance polyfunctional intra-tumoral CD8+ T cells and prevent rapid CD8+ T-cell exhaustion or energy, future efforts should focus on designing the optimal cancer vaccine constructs to guarantee the migration of these crucial immune cell subsets to their site of action. Positively, the KV heterologous prime-boost strategy described herein will be explored shortly in Stage IV colorectal cancer patients (NCT04046445).

## 5. Conclusions

In conclusion, we show that our heterologous prime-boost vaccination inducing cross-presenting cDC1s and CD4+ T cells convey a potent anti-tumor CD8+ T-cell response in both immune excluded and infiltrated mouse cancer models. This study also highlights the importance of the design of a multi-epitope vaccine for cancer immunotherapy.

## Figures and Tables

**Table 1:** CD4+ T cell % among CD45+ cells and Thelper subtype % among CD4+ T cells in the tumor or tumor-dLNs.

Tumor	Control	KK	KV	*p*-Value		
	Mean (±SD)			Ctrl vs. KK	Ctrl vs. KV	KK vs. KV
CD4 (%)	0.86 (0.79)	1.38 (0.59)	2.07 (0.53)	0.16	0.0003	0.04
CD4 (×10^5^)	1.33 (1.04)	8.67 (12.42)	10.7 (7.25)	0.11	0.03	0.83
Th1 (%)	14.84 (8.64)	31.22 (10.59)	51.62 (7.31)	0.0005	<0.0001	<0.0001
Th1 (×10^5^)	0.23 (0.28)	2.89 (3.61)	5.90 (4.21)	0.14	0.0007	0.09
Th2 (%)	23.95 (15.74)	21.98 (3.83)	10.15 (8.12)	0.74	<0.0001	0.0003
Th2 (×10^5^)	0.33 (0.26)	2.01 (2.67)	1.15 (1.08)	0.06	0.49	0.46
Th17 (%)	5.66 (3.72)	3.98 (2.16)	0.63 (0.64)	0.27	0.0002	0.01
Th17 (×10^5^)	0.07 (0.05)	0.44 (0.89)	0.07 (0.06)	0.24	1.00	0.23
Treg (%)	36.32 (6.16)	38.93 (7.83)	14.88 (8.38)	0.70	<0.0001	<0.0001
Treg (×10^5^)	0.58 (0.43)	3.48 (4.43)	1.78 (1.51)	0.054	0.56	0.32
**Lymph Nodes**	**Control**	**KK**	**KV**	***p*** **-Value**		
	**Mean (±SD)**			**Ctrl vs. KK**	**Ctrl vs. KV**	**KK vs. KV**
CD4 (%)	46.83 (2.94)	43.64 (4.14)	43.25 (3.31)	0.09	0.04	0.95
CD4 (×10^5^)	2.42 (1.23)	1.75 (1.02)	1.56 (0.61)	0.20	0.07	0.82
Th1 (%)	2.24 (1.68)	3.30 (1.97)	6.56 (3.38)	0.56	0.0004	0.002
Th1 (×10^5^)	0.03 (0.04)	0.06 (0.04)	0.10 (0.08)	0.34	0.01	0.11
Th2 (%)	2.01 (0.82)	1.90 (0.68)	1.16 (0.58)	0.91	0.01	0.01
Th2 (×10^5^)	0.03 (0.02)	0.04 (0.02)	0.02 (0.01	0.55	0.23	0.01
Th17 (%)	4.42 (6.53)	6.82 (5.64)	3.48 (2.42)	0.45	0.89	0.14
Th17 (×10^5^)	0.07 (0.13)	0.13 (0.11)	0.05 (0.05)	0.21	0.94	0.06
Treg (%)	7.49 (2.93)	10.23 (4.52)	11.85 (5.30)	0.30	0.054	0.56
Treg (×10^5^)	0,16 (0.07)	0,15 (0.05)	0,16 (0.07)	0.93	0.97	0.75

*n* = 11 per group for tumor control, KK and KV and *n* = 10 for control LNs, *n* = 17 for KK and KV LNs. *p*-values derived from One-Way ANOVA followed by Tukey’s multiple comparison test.

## Data Availability

Data are available on request due to restrictions, e.g., privacy or ethics. The data presented in this study are available on request from the corresponding author.

## Supplementary Materials

The following are available online at https://www.mdpi.com/article/10.3390/cancers13236107/s1, Figure S1. Tumor growth curves, intratumoral NK and NKT cells, intratumoral and intranodal dendritic cell characterization and immune cell ratios in TC-1 tumor-bearing C57BL/6 mice, Figure S2. CD8+ T-cell characteristics in TC-1 tumor-bearing C57BL/6 mice. Tumor growth curves, intratumoral NK and NKT cells, intratumoral and intranodal dendritic cell in MC-38 tumor-bearing C57BL/6 mice, Figure S3. CD4+ T-cell characterization of tumor and tumor-dLN and blood Ag correlation graphs in MC-38 tumor-bearing C57BL/6 mice, Figure S4. TIL characterization and Tcf1+/PD-1+ CD8+ T-cell stemness gating strategies, Figure S5. CD4+ T-helper and CD4+ regulatory T-cell gating strategies, Figure S6. DC characterization gating strategies, Figure S7. Cytokine concentrations from tumor and tumor-dLNs of TC-1 tumor-bearing C57BL/6 mice. Table S1. Mean values and statistical analysis of TIL characterization from Figure 4C, Table S2. Antibodies and Co-Stimulators, Table S3. Multimers.
