# Supplementary material for: Heterologous Prime-Boost Vaccination with a Peptide-Based Vaccine and Viral Vector Reshapes Dendritic Cell, CD4+ and CD8+ T Cell Phenotypes to Improve the Antitumor Therapeutic Effect
Source: Cancers (Basel). 2021 Dec 3;13(23):6107. doi: 10.3390/cancers13236107 (PMC8656755; doi:10.3390/cancers13236107)
Supplement: Supplementary file 1 [file cancers-13-06107-s001.zip › cancers-1470583-supplementary.pdf]

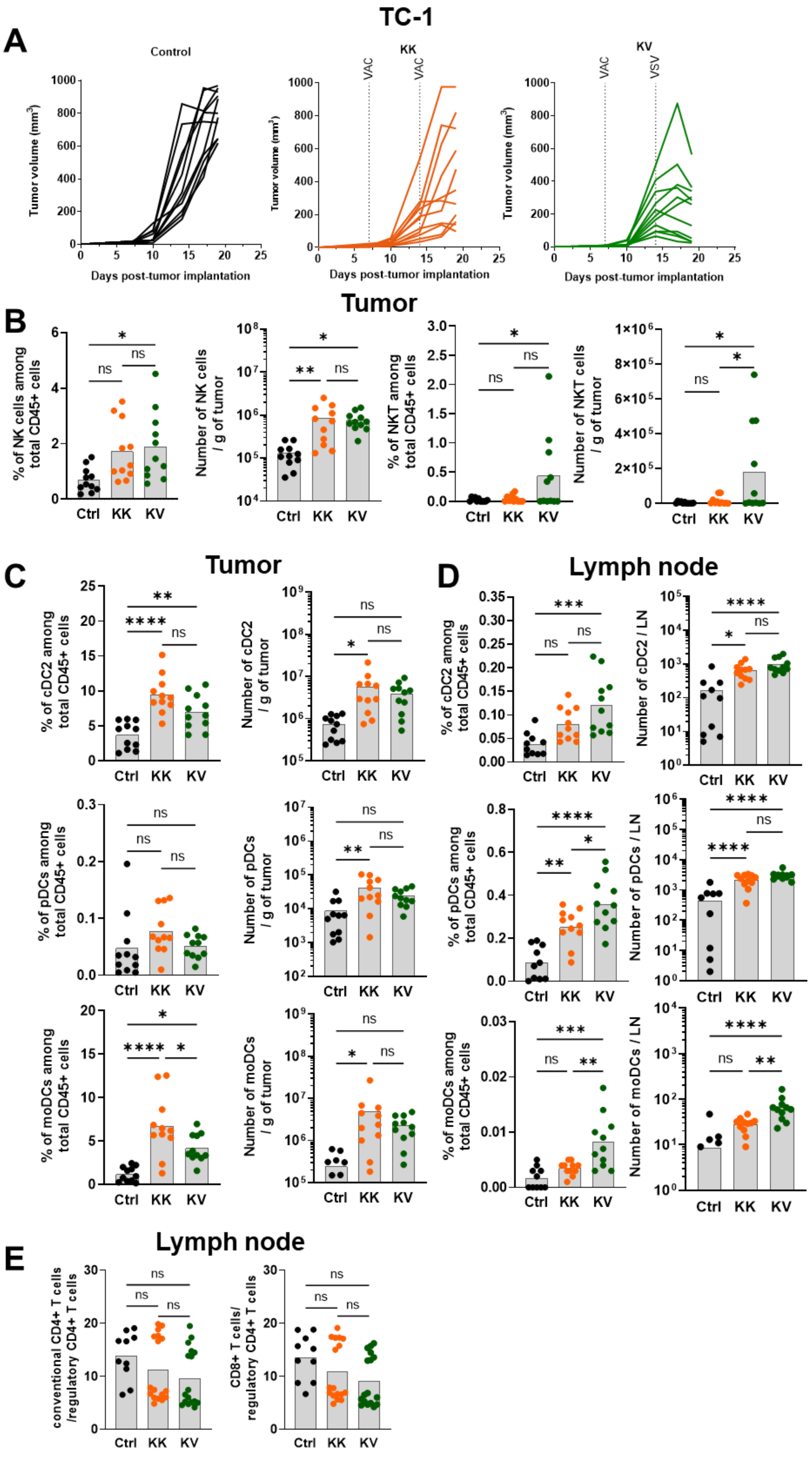

TC-1
A
Control
KK
KV
VAC
VSV
Tumor volume (mm3)
Days post-tumor implantation
B
Tumor
% of NK cells among total CD45+ cells
Number of NK cells / g of tumor
% of NKT among total CD45+ cells
Number of NKT cells / g of tumor
Ctrl KK KV
C
D
Lymph node
% of cDC2 among total CD45+ cells
Number of cDC2 / g of tumor
Number of cDC2 / LN
% of pDCs among total CD45+ cells
Number of pDCs / g of tumor
Number of pDCs / LN
% of moDCs among total CD45+ cells
Number of moDCs / g of tumor
Number of moDCs / LN
E
conventional CD4+ T cells /regulatory CD4+ T cells
CD8+ T cells/ regulatory CD4+ T cells
ns

Figure S1. Tumor growth curves, intratumoral NK and NKT cells, intratumoral and intranodal dendritic cell characterization and immune cell ratios in TC-1 tumor-bearing C57BL/6 mice. (**A**) Individual TC1 tumor-growth curves in mm^3 post-tumor implantation for untreated (control), homologous KISIMA-Mad25-treated (KK) and heterologous KISIMA-Mad25- and VSV-GP-HPV-treated (KV) groups up to two days before tumor harvest for FACS analysis. (**B**) Frequencies and absolute numbers of tumor-infiltrating NK (left) and NKT (right) cells were analyzed on day 21 post-TC-1 tumor implantation. (**C**) From top to bottom: Frequencies (left) and absolute numbers (right) of tumor-infiltrating cDC2s, pDCs and moDCs on day 18 post-MC-38 tumor implantation. (**D**) From top to bottom: Frequencies (left) and absolute numbers (right) of intranodal cDC2s, pDCs and moDCs on day 18 post-MC-38 tumor implantation. (**E**) Ratio of intranodal Tconv to Treg (left side) or intranodal CD8+ T cells to Treg derived from TC-1 tumor-bearing mice on day 21 post-tumor implantation. Data in (**A**) to (**E**) are shown as means (grey bar) and are derived from at least two independent experiments. * $p < 0.05$, ** $p < 0.01$, *** $p < 0.001$, **** $p < 0.0001$ One-Way ANOVA followed by Tukey's multiple comparison test.

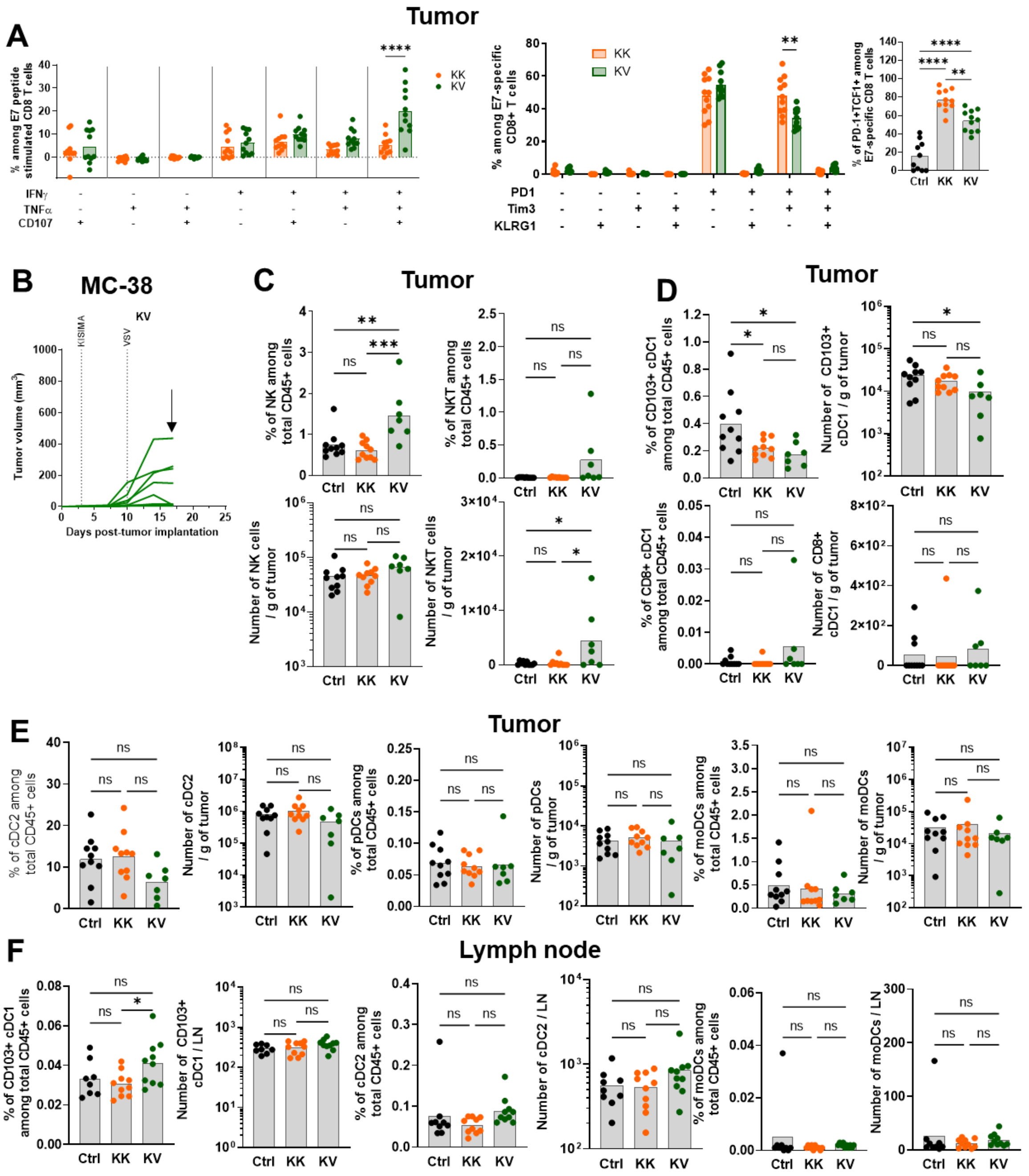

Tumor
A
% among E7 peptide stimulated CD8 T cells
KK
KV
IFNγ
TNFα
CD107
% among E7-specific CD8+ T cells
PD1
Tim3
KLRG1
% of PD-1+TCF1+ among E7-specific CD8 T cells
Ctrl
KK
KV
B
MC-38
KV
KISIMA
VSV
Tumor volume (mm³)
Days post-tumor implantation
C
Tumor
% of NK among total CD45+ cells
% of NKT among total CD45+ cells
Number of NK cells / g of tumor
Number of NKT cells / g of tumor
D
Tumor
% of CD103+ cDC1 among total CD45+ cells
Number of CD103+ cDC1 / g of tumor
% of CD8+ cDC1 among total CD45+ cells
Number of CD8+ cDC1 / g of tumor
E
Tumor
% of cDC2 among total CD45+ cells
Number of cDC2 / g of tumor
% of pDCs among total CD45+ cells
Number of pDCs / g of tumor
% of moDCs among total CD45+ cells
Number of moDCs / g of tumor
F
Lymph node
% of CD103+ cDC1 among total CD45+ cells
Number of CD103+ cDC1 / LN
% of cDC2 among total CD45+ cells
Number of cDC2 / LN
% of moDCs among total CD45+ cells
Number of moDCs / LN

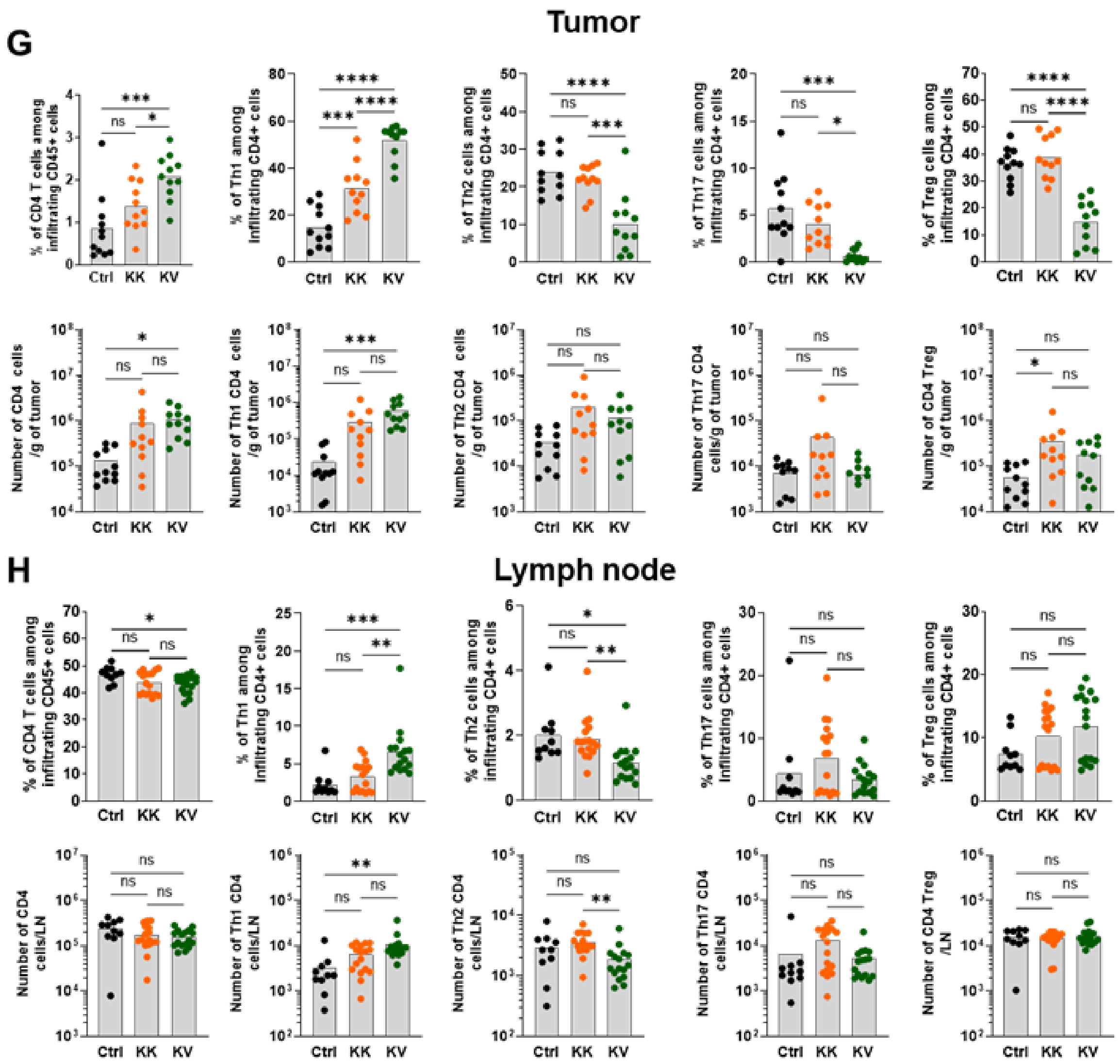

Figure S2. CD8+ T-cell characteristics in TC-1 tumor-bearing C57BL/6 mice. Tumor-growth curves, intratumoral NK and NKT cells, intratumoral and intranodal dendritic cell in MC-38 tumor-bearing C57BL/6 mice. (**A**) Left: Tumor-infiltrating CD8+ T cells were stimulated for 6 hours with E7 peptide and stained for IFN-γ, TNF-α and CD107α. Middle: The exhaustion status was examined on tumor-infiltrating E7-tetramer-positive CD8+ T cells in regards to expression of PD-1, Tim-3 and the effector marker KLRG1. Right: Tumor-derived E7-specific CD8+ T cells were stained for the co-expression of PD-1 and Tcf-1. Tumors were harvested on day 21 post-TC-1 tumor implantation. (**B**) Individual MC-38 tumor-growth curves in mm^3 post-tumor implantation for heterologous KISIMA-Mad46- and VSV-GP-Mad46-treated (KV) groups two days before tumor harvest (arrow) for FACS analysis. (**C**) Frequencies (top) and absolute numbers (bottom) of tumor-infiltrating NK (left) and NKT (right) cells were analyzed on day 18 post-MC-38 tumor implantation. (**D**) Frequencies (left) or absolute numbers (right) of either migratory CD103+ cDC1s (upper graphs) or tissue-resident CD8+ cDC1s (lower graphs) derived from inguinal lymph nodes on day 18 post-MC-38 tumor implantation. (**E**) From left to right: Frequencies and absolute numbers of tumor-infiltrating cDC2s, pDCs and moDCs on day 18 post-MC-38 tumor implantation. (**F**) From left to right: Frequencies and absolute numbers of intranodal migratory CD103+ cDC1s, cDC2s and moDCs on day 18 post-MC-38 tumor implantation. (**G**) From left to right: Frequencies and absolute numbers of tumor-infiltrating CD4 T cells, Th1, Th2, Th17 and Tregs on day 18 post-MC-38 tumor implantation. (**H**) From left to right: Frequencies and absolute numbers of intranodal CD4 T cells, Th1, Th2, Th17 and Tregs on day 18 post-MC-38 tumor implantation. Data in (**A**) to (**H**) are shown as means (grey bar) and are derived from at least two independent experiments. * $p < 0.05$, ** $p < 0.01$, *** $p < 0.001$, **** $p < 0.0001$ One-Way ANOVA followed by Tukey's multiple comparison test.

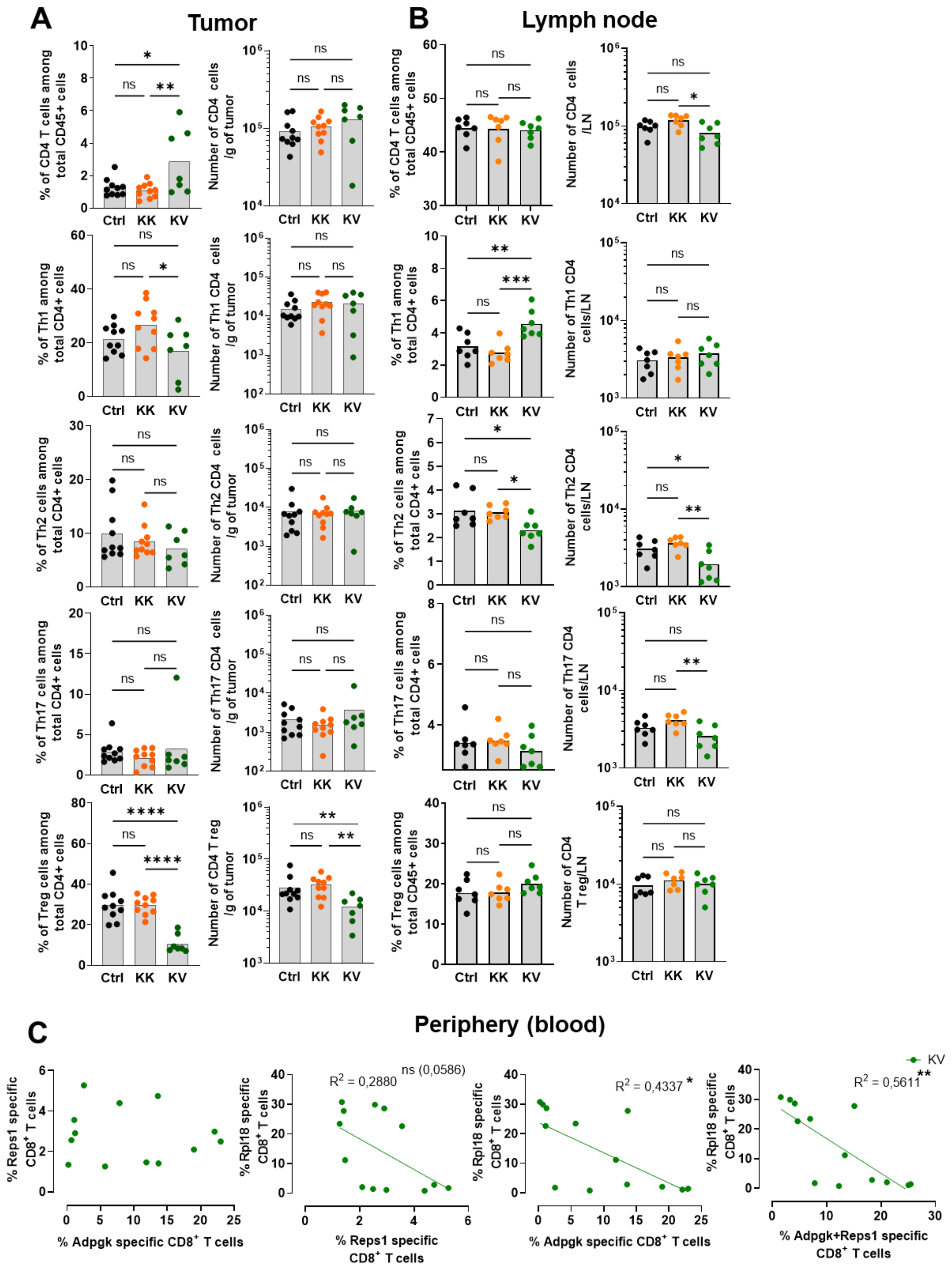

Figure S3. CD4+ T cell characterization of tumor and tumor-dLN and blood Ag correlation graphs in MC-38 tumor-bearing C57BL/6 mice. (**A**) From top to bottom: Frequencies (left) or numbers (right) of intratumoral CD4+ T cells, CD4+ Th1 cells, CD4+ Th2 cells, CD4+ Th17 cells or CD4+ Treg cells on day 18 post-MC-38 tumor implantation. (**B**) From top to bottom: Frequencies (left) or numbers (right) of intranodal CD4+ T cells, CD4+ Th1 cells, CD4+ Th2 cells, CD4+ Th17 cells or CD4+ Treg cells on day 18 post-MC-38 tumor implantation. (**C**) From left to right: Correlation graphs (KV-treated mice) of blood-derived Reps1-specific CD8+ T cell % to Adpgk-specific CD8+ T cell %, blood-derived Rpl18-specific CD8+ T cell % to Reps1-specific CD8+ T cell %, blood-derived Rpl18-specific CD8+ T cell % to Adpgk-specific CD8+ T cell % and blood-derived Rpl18-specific CD8+ T cell % to the sum of Reps1- and Adpgk-specific CD8+ T cell % on day 17 post-MC-38 tumor implantation. Data in (**A**) and (**B**) are shown as means (grey bar), (**A**) is derived from two independent experiments and (**B**) from one experiment. * $p < 0.05$, ** $p < 0.01$, *** $p < 0.001$, **** $p < 0.0001$ One-Way ANOVA followed by Tukey's multiple comparison. Data in C) are derived from two independent experiments. * $p < 0.05$, ** $p < 0.01$ are calculated with simple linear regression.

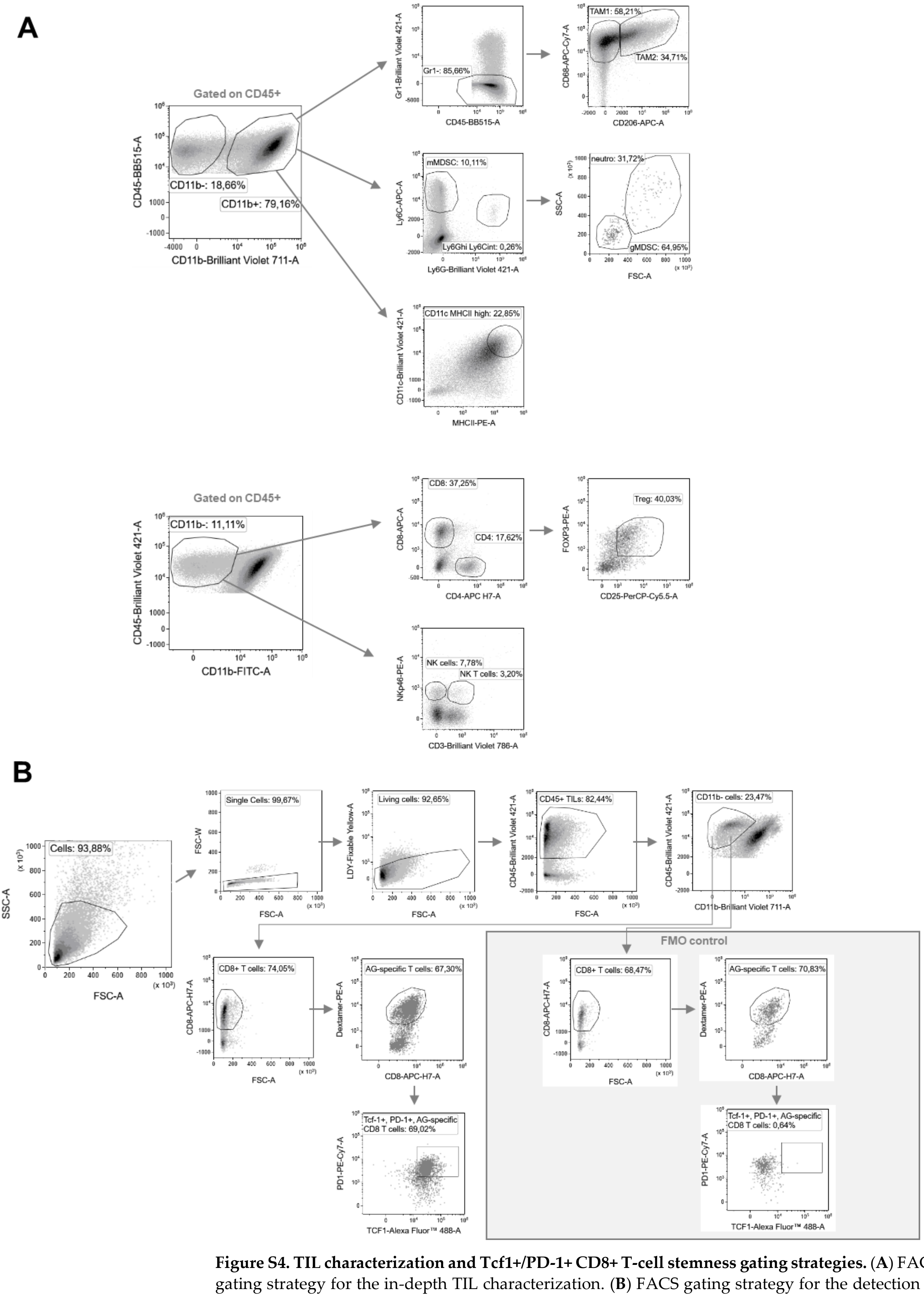

Figure S4. TIL characterization and Tcf1+/PD-1+ CD8+ T-cell stemness gating strategies. (**A**) FACS gating strategy for the in-depth TIL characterization. (**B**) FACS gating strategy for the detection of Tcf-1+/PD-1+ antigen-specific CD8+ T cells.

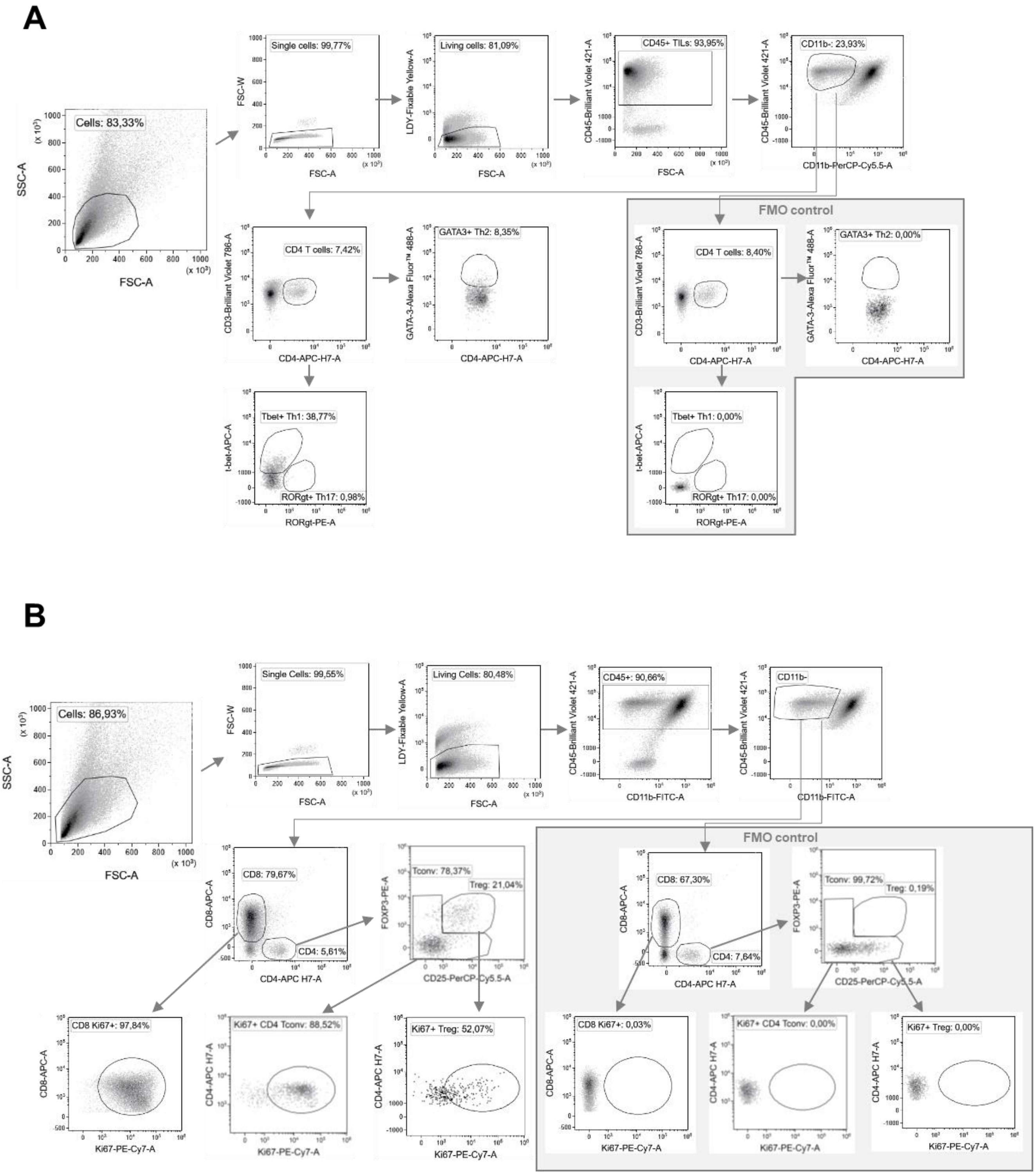

Figure S5. CD4+ T-helper and CD4+ regulatory T-cell gating strategies. (**A**) FACS gating strategy for the characterization of Th1, Th2 and Th17 CD4+ cells. (**B**) FACS gating strategy for the detection of CD4+ regulatory T cells and the investigation of proliferation via the proliferation marker Ki67.

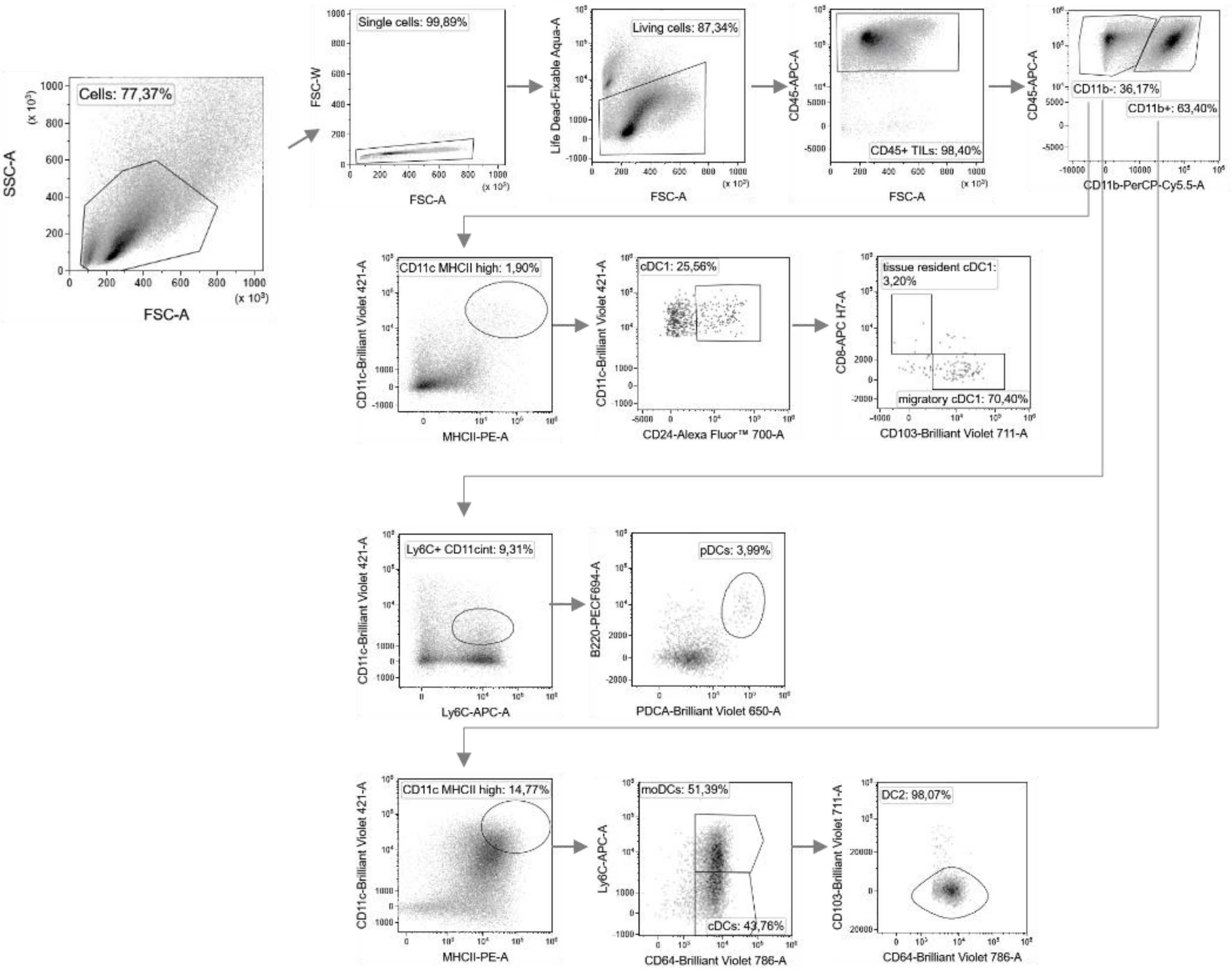

Figure S6. DC characterization gating strategies. FACS gating strategies for the characterization of tissue-resident CD8+ cDC1s, migratory CD103+ cDC1s, moDCs, cDC2s and pDCs.

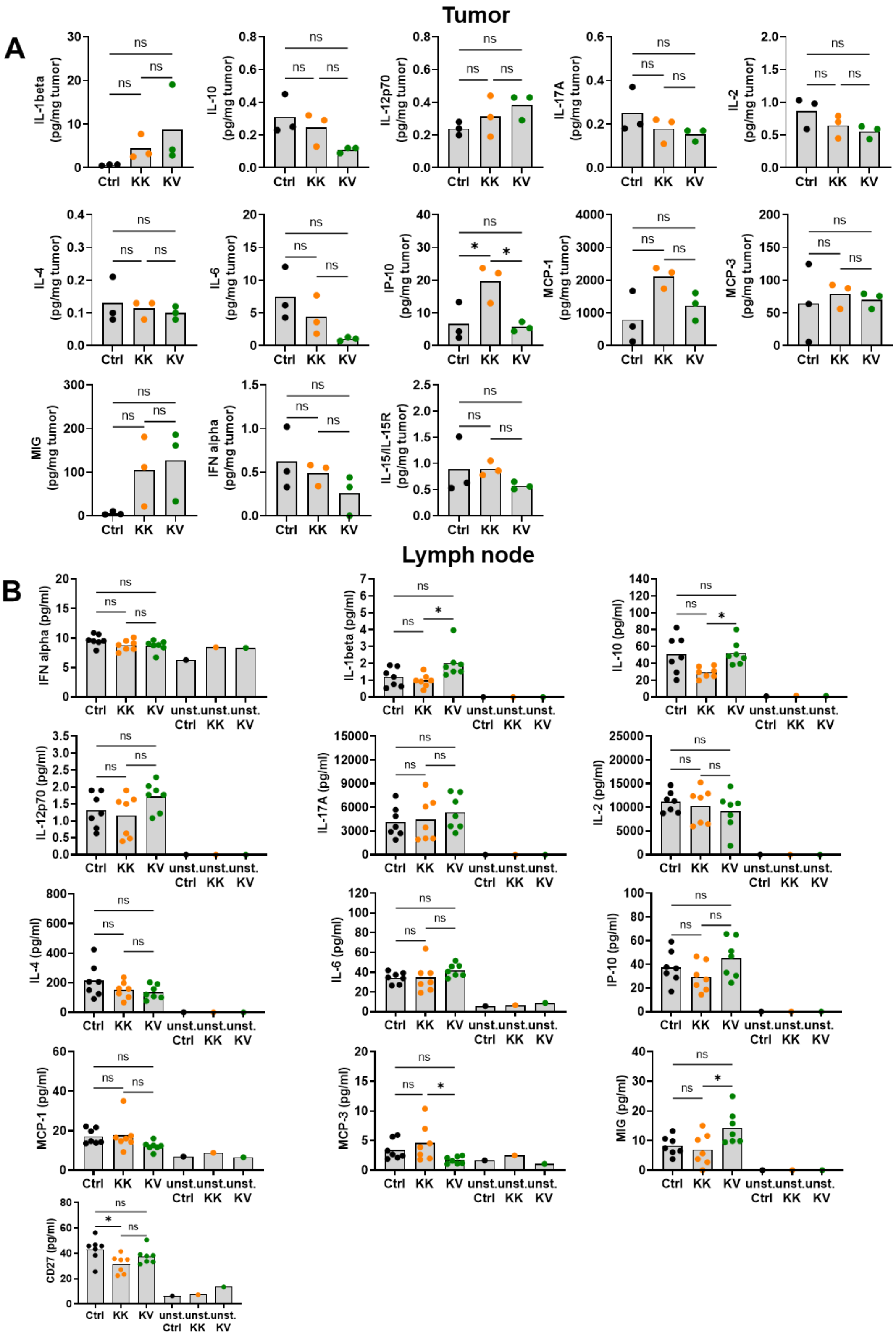

Figure S7. Cytokine concentrations from tumor and tumor-dLNs of TC-1 tumor-bearing C57BL/6 mice. (**A**) Cytokine (IL-1beta, IL-10, IL-12p70, IL-17A, IL-2, IL-4, IL-6, IP-10, MCP-1, MCP-3, MIG, IFN-α, IL-15/IL-15R) concentrations in pg/mL derived from tumor lysates that were harvested on day 21 post-tumor implantation and measured by Luminex. (**B**) Cytokine (IFN-α, IL-1beta, IL-10, IL-12p70, IL-17A, IL-2, IL-4, IL-6, IP-10, MCP-1, MCP-3, MIG, CD27) concentrations in pg/mL derived from inguinal lymph node cell suspension supernatants 24 hours after re-stimulation with α-CD3/α-CD28 beads or respective controls, which were harvested on day 21 post-tumor implantation and measured by Luminex. Data in (**A**) and (**B**) are shown as means (grey bar) and are derived from one experiment. * $p < 0.05$ One-Way ANOVA followed by Tukey's multiple comparison test.

Table S1. Mean values and statistical analysis of TIL characterization from Figure 4C on day 18 post-MC-38 tumor implantation. Statistical analysis was performed using One-Way ANOVA followed by Tukey's multiple comparison test.

Tumor	Mean % (±SD)			*p*-value		
	Ctrl	KK	KV	Ctrl vs KK	Ctrl vs KV	KK vs KV
**CD8+ T cells**	3.6 (2.6)	4.3 (1.9)	42.6 (23.6)	0.9893	**<0.0001**	**<0.0001**
**CD4+ Tregs**	0.4 (0.3)	0.4 (0.13)	0.36 (0.37)	0.991	0.9274	0.965
**CD4+ Tconv**	1.04 (0.17)	0.93 (0.18)	2.75 (1.63)	0.9503	**0.001**	**0.0005**
**mMDSCs**	9.5 (4.8)	7.3 (4.1)	5.22 (3.15)	0.4661	0.1123	0.5793
**gMDSCs**	0.38 (0.37)	0.15 (0.12)	0.19 (0.24)	0.1646	0.3499	0.9526
**Neutrophils**	0.22 (0.18)	0.08 (0.04)	0.05 (0.04)	**0.0309**	**0.0188**	0.8837
**TAM1**	33.3 (13.1)	42.3 (11.7)	20.7 (12.7)	0.2636	0.1248	**0.0052**
**TAM2**	41.5 (13.9)	35.7 (13.1)	24.2 (12.4)	0.5973	**0.0371**	0.2083
**NKT**	0 (0)	0 (0)	0.27 (0.47)	0.9999	0.0672	0.0672
**NK**	0.74 (0.33)	0.61 (0.22)	1.46 (0.67)	0.7568	**0.0046**	**0.0009**
**B**	0.46 (0.25)	0.36 (0.21)	0.68 (0.91)	0.9035	0.6597	0.4268
**DCs**	12.8 (6.3)	13.2 (6.5)	6.98 (4.39)	0.9857	0.1372	0.1042

Table S2. Antibodies and Co-Stimulators.

Antibody specificity	Clone	Manufacturer
CD45	30-F11	BD
CD11b	M1/70	BD
PD1	29F.1A12	Biolegend
CD8	53-6.7	BD
KLRG1	2F1	BD
Tim3	B8.2C12	Biolegend
CD103	M290	BD
CD49a	Ha31/8	BD
Ly6C	AL-21	BD
Ly6G	1A8	BD
PD-L1	10F.9G2	Biolegend
CD103	M290	BD
MHCII	2D4	BD
CD11c	HL3	BD
CD40	HM40-3	eBioscience
B220	RA3-6B2	BD
CD24	M1/69	BD
CD64	X54-5/7.1	BD
Pdca	927	BD
CD4	RM4-5	BD
CD4	GK1-5	BD
CD25	PC61.5	TONBO Bioscienes
FoxP3	FJK-16s	eBioscience
Ki67	solA15	eBioscience
Gr1	RB6-8C5	BD
LAP (TGF-beta)	TW 7-16B4	BioLegend
CD3	500A2	BD
CD335 (NKp46)	29A1.4	BD
CD206	C068C2	BioLegend
CD68	Fa-11	BioLegend
T-bet	eBio4B10 (4B10)	eBioscience
GATA-3	TWAJ	eBioscience
RORgt	AFKJS-9	eBioscience
CD69	H1.2F3	BD
TNFa	MP6-XT22	BD
IFNg	XMG1.2	BD
IL-2	JES6-5H4	BD
Isotype rat IgG1k	R3-34	BD
Isotype rat IgG1k	G234-2356	BD
Isotype rat IgG2bk	A95-1	BD
Anti-mouse CD28 (for co-stimulation)	Mouse EL-4 (T-cell lymphoma Cells)	BD
Anti-mouse CD3e (for co-stimulation)	H-2Kb-specific cytotoxic T lymphocyte clone BM10-37	BD

Table S3. Multimers.

Multimers	Cat. N°	Manufacturer
E7 MHC dextramer (H-2Db RAHYNIVTF - PE)	JA2195-PE	Immudex
Adpgk MHC dextramer (H-2Db ASMTNMELM - PE)	JA3803-PE	Immudex
Reps1 MHC tetramer (H2-Db AQLANDVVL - PE)	TB-5114-1	MBL
Rpl18 MHC tetramer (H2-Kb KILTFDRL - PE)	MKb-017	Tetramer Shop
